# Comparing the Performance of Microsatellites and RADseq in Population Genetic Studies: Analysis of Data for Pike (*Esox lucius*) and a Synthesis of Previous Studies

**DOI:** 10.3389/fgene.2020.00218

**Published:** 2020-03-13

**Authors:** Johanna Sunde, Yeşerin Yıldırım, Petter Tibblin, Anders Forsman

**Affiliations:** Department of Biology and Environmental Science, Centre for Ecology and Evolution in Microbial Model Systems, EEMiS, Linnaeus University, Kalmar, Sweden

**Keywords:** adaptation, differentiation, *Esox lucius*, genetic structure, microsatellites, RADseq

## Abstract

Population genetic studies reveal biodiversity patterns and inform about drivers of evolutionary differentiation and adaptation, including gene flow, drift and selection. This can advance our understanding and aid decision making regarding management and conservation efforts. Microsatellites have long been used in population genetic studies. Thanks to the development of newer techniques, sequencing approaches such as restriction site associated DNA sequencing (RADseq) are on their way to replace microsatellites for some applications. However, the performance of these two marker types in population genetics have rarely been systematically compared. We utilized three neutrally and adaptively differentiated populations of anadromous pike (*Esox lucius*) to assess the relative performance of microsatellites and RADseq with respect to resolution and conclusiveness of estimates of population differentiation and genetic structure. To this end, the same set of individuals (*N* = 64) were genotyped with both RADseq and microsatellite markers. To assess effects of sample size, the same subset of 10 randomly chosen individuals from each population (*N* = 30 in total) were also genotyped with both methods. Comparisons of estimated genetic diversity and structure showed that both markers were able to uncover genetic structuring. The full RADseq dataset provided the clearest detection of the finer scaled genetic structuring, and the other three datasets (full and subset microsatellite, and subset RADseq) provided comparable results. A search for outlier loci performed on the full SNP dataset pointed to signs of selection potentially associated with salinity and temperature, exemplifying the utility of RADseq to inform about the importance of different environmental factors. To evaluate whether performance differences between the markers are general or context specific, the results of previous studies that have investigated population structure using both marker types were synthesized. The synthesis revealed that RADseq performed as well as, or better than microsatellites in detecting genetic structuring in the included studies. The differences in the ability to detect population structure, both in the present and the previous studies, are likely explained by the higher number of loci typically utilized in RADseq compared to microsatellite analysis, as increasing the number of markers will (regardless of the marker type) increase power and allow for clearer detection and higher resolution of genetic structure.

## Introduction

Investigations of population genetic structure reveal patterns of biological diversity and inform about the relative importance of underlying evolutionary drivers, such as gene flow, genetic drift and selection (Loiselle et al., 1995). For example, evidence of genetic differentiation among populations is indicative of restricted gene flow, and informs about geographical and/or ecological barriers to dispersal or gene flow (Yıldırım et al., 2018a; Nordahl et al., 2019). Strong genetic differentiation may also reflect divergent selection (Charlesworth and Charlesworth, 2017). Population genomic tools have the possibility to identify such putatively adaptive variation among populations, as genetic variation might be associated with locally adaptive phenotypes and divergent selection driven by environmental heterogeneity (Funk et al., 2012). Conversely, low genetic structuring among populations may indicate weak selection or result from high dispersal, metapopulation structures, or recent population divergence (Vendrami et al., 2017). Population genetic studies can thus provide insights into how the different processes have influenced the evolution of populations and species (Charlesworth and Charlesworth, 2017). Knowledge about population genetic structure is also essential when designing conservation efforts (e.g., translocations, supplementations and (re)-introductions) to ensure successful management (Stephenson, 1999; Hutchinson, 2008; Wright et al., 2015; Nordahl et al., 2019).

Different types of genetic markers have been widely used to delineate populations. One of the most frequently used marker types in studies of population structure is microsatellites, which are short tandem repeats (of 1–6 bp) in the DNA that are abundant in the genome of most taxa (Field and Wills, 1996). Most microsatellites behave as selectively neutral markers, but some have been found to be influenced by selection (e.g., in Cortazar-Chinarro et al., 2017), presumably because of their linkage to other loci that are under selection. Because of the high mutation rate of microsatellites, they are highly polymorphic markers that can inform about recent population divergence (Edwards and Bensch, 2009; Zink et al., 2013; Hodel et al., 2017), and have proven successful in detecting population structure even among closely related populations (e.g., Wei et al., 2013; Yıldırım et al., 2018a; Nordahl et al., 2019). Due to logistical constrains, microsatellite studies have traditionally used a relatively low number of markers (<25), which brings about a risk of underestimating genetic structure due to insufficient numbers of polymorphic loci (Hodel et al., 2017). When low numbers of genetic markers are used, larger sample sizes are required to generate accurate estimates of allele frequencies and diversity (e.g., Hale et al., 2012), which might be difficult to achieve, especially for endangered species with small population sizes. However, there is no intrinsic limitation to the number of microsatellite markers that can be used, and by increasing the number of markers included, the required sample size would decrease. To obtain higher numer of markers, recent studies have begun to utilize next-generation sequencing to genotype microsatellites, which has proven successful, and even hundreds of microsatellites have been used within studies (e.g., in Bradbury et al., 2018).

Thanks to technological advances in next-generation sequencing (NGS), alternative sequencing approaches for studying population genetics and genomics are becoming readily available, such as restriction site associated DNA sequencing (RADseq) methods (e.g., RAD-Tag; Baird et al., 2008; ddRAD-seq; Peterson et al., 2012; and 2b-RAD; Wang et al., 2012). RADseq targets sequences adjacent to common restriction sites distributed throughout the genome and may recover thousands of loci that can be used to search for single nucleotide polymorphisms (SNPs) and indels to investigate population structure and diversity (Andrews et al., 2016). RADseq is applicable to non-model organisms (Andrews et al., 2016), although it produces more satisfactory results when there is a reference genome available for alignment. Due to the slower mutation rate of SNPs on average, the proportion of recent alleles are lower, and each SNP is therefore typically less informative about recent population divergence than the microsatellite markers. Provided that this can be compensated for by a higher number of markers, it has been argued that RADseq can perform as well as, or even better than microsatellites in detecting population structure and divergence (e.g., Hodel et al., 2017; Morgan et al., 2017; Bohling et al., 2019), even for small datasets (Jeffries et al., 2016; Nazareno et al., 2017; Vendrami et al., 2017). It has also been argued that the slower mutation rate of SNPs makes them better suitable for revealing ancestral patterns of genetic structuring (Zhang and Hewitt, 2003; Andrews et al., 2016; but see Sun et al., 2009, for evidence to the contrary).

Both microsatellites and RADseq markers have potential to inform about the role of different evolutionary processes. Loci that are not genetically linked to coding or regulatory regions can inform about patterns of genetic variation that result from neutral or stochastic processes. In contrast, loci associated with coding regions can be used to quantify adaptive variation, and inform about functional deterministic processes such as selection. For example, it is possible to detect outlier loci that have putatively been influenced by selection and to search for associations with environmental factors that may impact genetic structure (de Villemereuil and Gaggiotti, 2015; Andrews et al., 2016), and by annotating the outlier loci, candidate genes with known functions can be identified (Hohenlohe et al., 2010; Nadeau et al., 2014; Brieuc et al., 2015; Pujolar et al., 2015).

Some difficulties in applicability of the RADseq method restricts its usage in some ecological and evolutionary studies. For example, preparation of RADseq libraries require high molecular weight DNA, and the use of highly degraded DNA result in dramatic decrease in the number of raw reads recovered, and the quality of the reads (Graham et al., 2015). The library preparation also requires relatively large amount of starting material (50–100 ng of DNA; Andrews et al., 2016), and if excluding PCR amplification the required amount is even higher (1–2 μg of DNA; Toonen et al., 2013). It is also worth noting that PCR amplification introduces biases such as unequal representation of individuals, PCR duplicates and allele drop-outs (Andrews et al., 2016). Therefore, the utility of RADseq are restricted in studies where only small amounts of DNA are available, for example in studies that uses DNA samples collected with non-invasive methods (such as fecal samples) or tissue samples from museums and archeological specimens. Microsatellites, on the other hand, can yield accurate genotyping with highly degraded DNA and low amount of starting material (e.g., Regnaut et al., 2006; Chassaing et al., 2018). Another disadvantage with the RADseq method is that, despite the decreasing cost of next generation sequencing, it still has a relatively higher cost per individual than microsatellites (Lemopoulos et al., 2019). Because multiallelic microsatellites are available for many species, utilization of them might be both less costly and laborious than RADseq, and the preferred marker type might differ depending on the aim(s) of the study (Lemopoulos et al., 2019).

**FIGURE 1:**
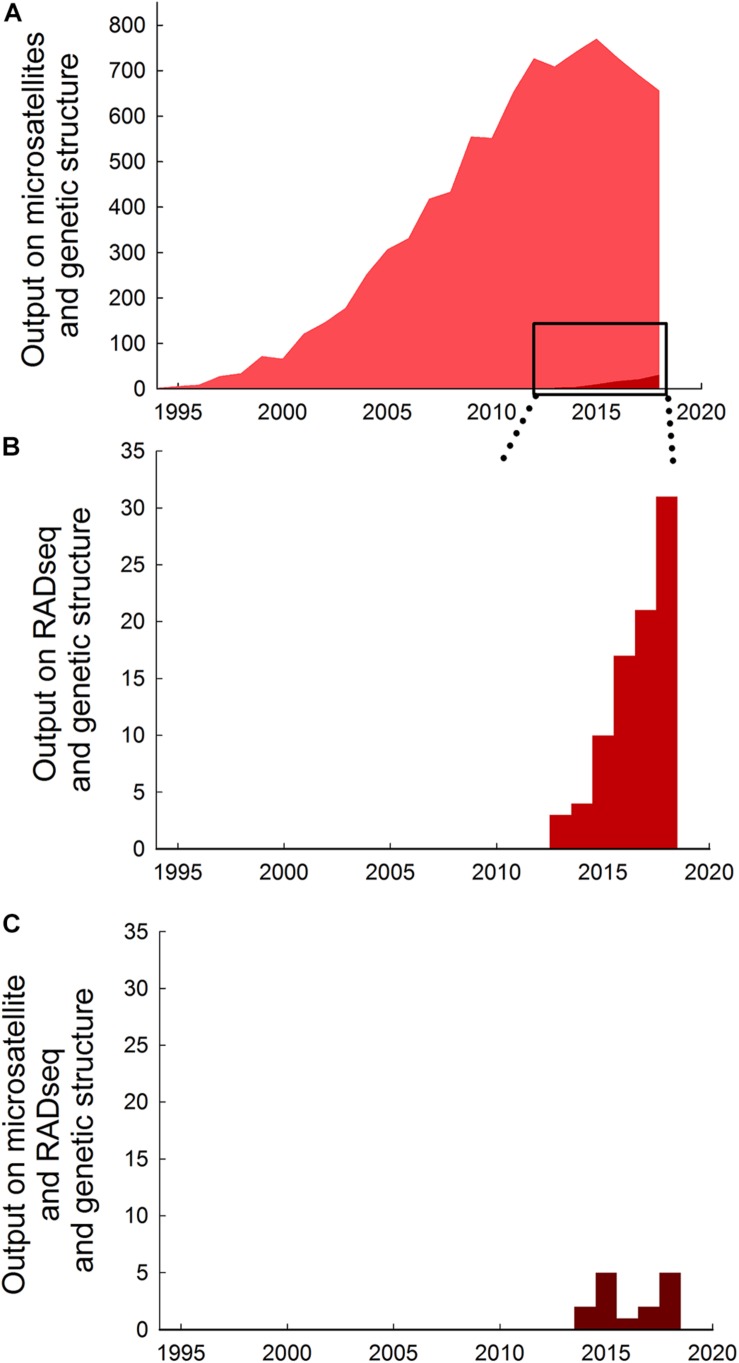
Trends in research output on the use of microsatellites and RADseq in studies of genetic structure. **(A)** Absolute research output measured as publications on microsatellites and genetic structure published per year up to December 2018^1^. **(B)** Absolute research output on RADseq and genetic structure per year up to December 2018^2^. **(C)** Absolute research output on microsatellites and RADseq and genetic structure per year up to December 2018^3^. Note that vertical axes are different in the different panels. Data extracted from a topic search conducted 19 June 2019 in ISI Web of Science (Data base: core collection; Time span: 1900-2018) using the following search strings: ^1^ (microsatellite^∗^ OR micro-satellite^∗^) AND (“genetic structur^∗^”), generated 9185 papers. ^2^ (RADseq OR Rad-seq) AND (“genetic structur^∗^”), generated 86 papers. ^3^ (microsatellite^∗^ OR micro-satellite^∗^) AND (RADseq OR Rad-seq) AND (“genetic structur^∗^”), generated 15 papers. ^1^ (microsatellite^∗^ OR micro-satellite^∗^) AND (“genetic structur^∗^”), generated 9185 papers. ^2^ (RADseq OR Rad-seq) AND (“genetic structur^∗^”), generated 86 papers. ^3^ (microsatellite^∗^ OR micro-satellite^∗^) AND (RADseq OR Rad-seq) AND (“genetic structur^∗^”), generated 15 papers.^1^ (microsatellite^∗^ OR micro-satellite^∗^) AND (“genetic structur^∗^”), generated 9185 papers. ^2^ (RADseq OR Rad-seq) AND (“genetic structur^∗^”), generated 86 papers. ^3^ (microsatellite^∗^ OR micro-satellite^∗^) AND (RADseq OR Rad-seq) AND (“genetic structur^∗^”), generated 15 papers.^1^ (microsatellite^∗^ OR micro-satellite^∗^) AND (“genetic structur^∗^”), generated 9185 papers. ^2^ (RADseq OR Rad-seq) AND (“genetic structur^∗^”), generated 86 papers. ^3^ (microsatellite^∗^ OR micro-satellite^∗^) AND (RADseq OR Rad-seq) AND (“genetic structur^∗^”), generated 15 papers.

RADseq has gained popularity and represent a valuable alternative to microsatellite markers for population genetic analyses (Andrews et al., 2016). Use of microsatellites in studies of genetic structure has grown tremendously over the past 30 years from less than 5 papers per year prior to 1995 to nearly 800 papers in 2015, but output has declined thereafter to about 650 papers per year in 2018 (Figure 1A), coinciding with the development of newer techniques. The number of studies using RADseq methods to investigate genetic structure is still comparatively low (<1% compared to microsatellites in total, and <10% compared to microsatellites in 2018), but has increased sixfold from about 5 papers per year prior to 2014 to >30 papers per year in 2018 (Figure 1B).

In view of the current development, it seems important to systematically evaluate and compare the utility and relative performance of the two different marker types when applied to the same set of sampling populations and individuals. Given that knowledge and understanding of genetic structure is required for informed decision making pertaining to protection and utilization of biodiversity (Stephenson, 1999; Hutchinson, 2008; Wright et al., 2015; Nordahl et al., 2019), it is also necessary to investigate whether any differences in the relative performance (e.g., resolution and conclusiveness of estimates of population genetic structure) of microsatellites and RADseq markers are context specific or consistent across species, populations, and environmental settings.

**FIGURE 2:**
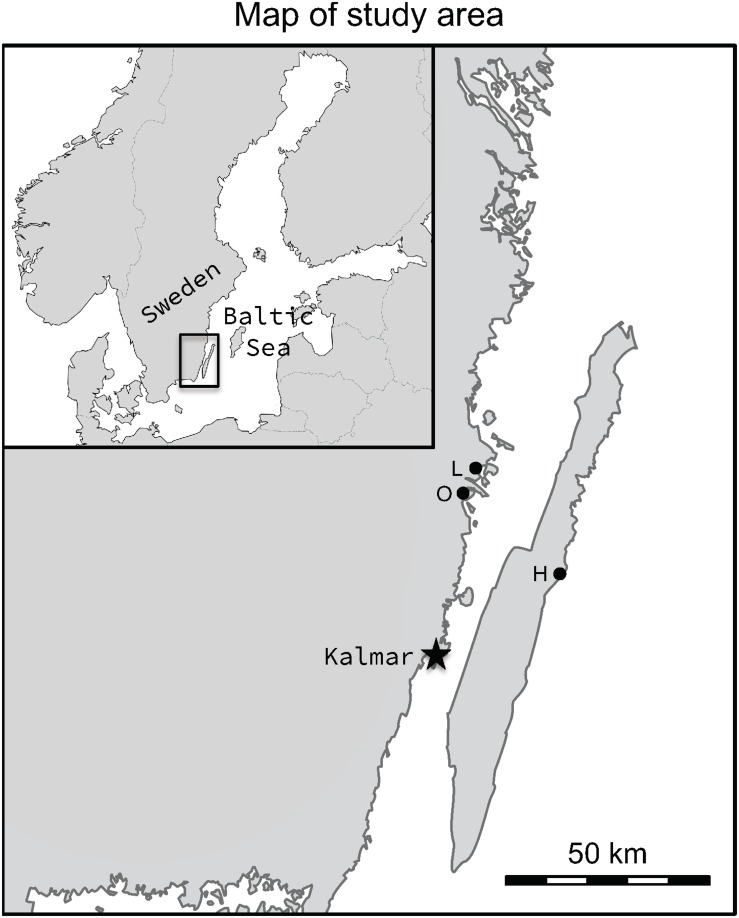
Map showing the locations of the study populations, Lerviksbäcken (L), Oknebäcken (O), and Harfjärden (H). The map was created in Adobe Photoshop CC, v. 2015.0.1, by combining and modifying two base maps, one of Scandinavia and one of Sweden, which are available under non-restrictive creative commons license obtained from Wikimedia Commons, https://commons.wikimedia.org/wiki/File:Scandinavia-template.png and https://commons.wikimedia.org/wiki/File:Sweden_location_map.svg.

In this study, we compared the performance of microsatellites and RADseq in detecting genetic structuring among three populations of anadromous pike (*Esox lucius*) in the Kalmar Sound region of the Baltic Sea (for sampling locations see Figure 2). In the Kalmar Sound, where individuals were sampled for this study, anadromous pike have a homing behavior (Engstedt et al., 2014; Larsson et al., 2015; Tibblin et al., 2016b) and return to their natal freshwater spawning ground for reproduction (Muller, 1986; Engstedt et al., 2010, 2014). Reproductive isolation resulting from natal site fidelity, in combination with differences in selective regimes among spawning locations has resulted in phenotypic differentiation in salinity tolerance (Sunde et al., 2018a), temperature tolerance (Sunde et al., 2019), vertebral number (Tibblin et al., 2016a), adult body size and growth rate (Tibblin et al., 2015), and early life history traits and reproductive investment (Berggren et al., 2016) even among closely located populations (including the ones sampled in the present study). This divergence in both neutral markers and functional traits makes them particularly suitable for a comparative study of RADseq and microsatellites, and to the best of our knowledge, this is the first study where RADseq has been implemented in studying genetic structure of *E. lucius*. To this end, we genotyped the same set of 64 individuals from the three populations (21–22 individuals from each population) using both methods (10 microsatellite loci and 1580 SNPs derived from RADseq). In addition, both microsatellite and RADseq genotyping was performed on the same subset of 10 randomly chosen individuals from each population to evaluate whether and how sample size influenced resolution or conclusiveness of the estimates of population genetic structure. The obtained data were first used to investigate and compare population genetic structure and connectivity (gene flow) among the populations as estimated by both marker types and for the different sample sizes. Next, we utilized the RADseq data to search for loci under selection to identify candidate genes putatively responsible for the phenotypical variation observed among the populations. We also investigated whether selection was correlated with two environmental variables (salinity and temperature) to which these populations have been found to be locally adaptated (Sunde et al., 2018a, 2019). Thereafter, to further evaluate the relative performance of microsatellite and RADseq markers, we conducted a literature search to identify previous studies that have used both marker types to study genetic structure, and compared, summarized, and synthesized the findings and conclusions of the previous studies.

## Materials and Methods

### Study Species

*Esox lucius* is a large predatory fish that plays an important role in many aquatic ecosystems by exerting top-down control (Donadi et al., 2017), i.e., regulating abundances of species in lower trophic levels. It is also economically important, as a valued species for both recreational and commercial fishing (Pierce et al., 1995; Lehtonen et al., 2009), and an important model species in studies of ecology and evolution (Forsman et al., 2015). Unfortunately, declines in pike populations in the Baltic Sea have been observed during the last decades (Lehtonen et al., 2009; Ljunggren et al., 2010; Nilsson et al., 2019; Olsson, 2019), and management actions, e.g., restoration of spawning habitats, have therefore been conducted to support the populations (Larsson et al., 2015). Population assignments that could be used to aid in decisions concerning such management efforts have mainly been based on studies utilizing neutral microsatellite markers (Bekkevold et al., 2015; Wennerström et al., 2016; Eschbach et al., 2019; Nordahl et al., 2019).

### Study Localities and Sampling Procedure

In the present study, three subpopulations of anadromous pike (Harfjärden, Lerviksbäcken and Oknebäck; henceforth Harfjärden, Lervik and Okne) that spawn in the Southeastern part of Sweden were included (Figure 2). Two of the subpopulations spawn in the Southeastern part of the Swedish mainland (Lervik: N 57° 04.414′; E 16° 31.246′, and Okne: N 57° 01.200′; E 16° 26.700′), and are separated by as little as approximately 20 km (shortest swimming distance). The third subpopulation spawns on the East coast of the island of Öland (Harfjärden: N 56° 49.063′; E 16° 48.673′), and is separated from the other two subpopulations by 120 km and 135 km respectively (shortest swimming distance).

In total, 64 individuals from the three populations (Harfjärden *N*_females_ = 12, *N*_males_ = 10, Lervik *N*_females_ = 9, *N*_males_ = 12, Okne *N*_females_ = 11, *N*_males_ = 10) were captured using fyke nets placed in the inlet of the streams leading up to the spawning locations during 4 days of the spawning migration in the spring of 2016 (between March 31 and April 5; for details see Sunde et al., 2018a, b). Non-lethal sampling of DNA (fin clip) were conducted for each individual, and the individuals were then released back into the water. The DNA samples were immediately placed in separate 1.5 mL Eppendorf tubes with 70% ethanol and kept on ice until transported to the Kalmar Sound Laboratory of Linnaeus University in Kalmar, Sweden, where they were subsequently stored in a freezer (−20°C) until the molecular work was conducted. The sample of individuals from the three populations in the present study are different from the sample of individuals representing the same populations in the study by Nordahl et al. (2019).

### Molecular Workflow

#### DNA Extraction and Microsatellite Genotyping

Tissue from each individual was ground using stainless steel beads (Next Advance, United States) and a Bullet Blender, and DNA was extracted using DNeasy blood and tissue kit (Qiagen, United States) according to the manufacturer’s instructions. All 64 samples were genotyped for 10 microsatellite loci (Elu 2, Elu 6, Elu 19, Elu 37, Elu 51, Elu 64, Elu 76, Elu 78, Elu 87, and Elu 276; Miller and Kapuscinski, 1996, 1997; Hansen et al., 1999), using Extract-N-Amp Blood PCR Kit for PCR amplification. Fragment analysis was performed by the Uppsala Genome Center (Uppsala, Sweden) using the size standard GeneScan^TM^ 500 ROX^TM^ (Thermo Fisher Scientific), and alleles were scored in the Microsatellite Plugin 1.4.4 in Geneious^®^ Pro 11.0.3 (Biomatters Ltd., New Zealand).

#### RADseq Library Preparation and Sequencing

For RADseq, the extracted DNA was digested with HF *Eco*RI (New England Biolabs, United States) at 37°C for 16 h. Size-selection of the fragments, paired-end library construction and sequencing was conducted by Science for Life Laboratory (Stockholm, Sweden; henceforth SciLifeLab). The 64 samples used in this study were sequenced along with additional samples (166 samples in total) on two lanes of Illumina HiSeq2500 with a 2 × 126 bp setup, which yielded a total of 89.1 M raw reads for the 64 samples (mean 1.4 M per sample).

#### Quality Control and Genotyping

Demultiplexing, quality control (with MultiQC; Ewels et al., 2016) and trimming of raw reads (with Trimmomatic; Bolger et al., 2014) to remove adapters and to truncate the reads to 100 bp (because uniform length of the reads is required for the Stacks pipeline) were performed by SciLifeLab. Out of the 89.1 M raw reads 79.7 M reads (89.4%) were kept after quality control and trimming (mean 1.2 M trimmed reads per sample), which were subsequently used for SNP discovery. *De novo* assembly, SNP calling, alignment to the reference genome and annotation were performed using the integrated approach of the Stacks *de novo* pipeline (Paris et al., 2017). In order to choose appropriate values for the parameters *m* (minimum number of raw reads required to form a stack/putative allele), *M* (number of mismatches allowed between stacks/putative alleles to merge them into a putative locus), and *n* (number of mismatches allowed between stacks/putative loci during construction of the catalog) to use in the Stacks pipeline (Catchen et al., 2011, 2013), we first performed parameter optimization on a subset of the individuals (10 individuals from each population) as described in Paris et al. (2017) and Rochette and Catchen (2017). The results from the parameter optimization suggested to use *m* = 3, *M* = 3, and *n* = 3 (for details see section Parameter Optimization and Supplementary Figures S1–S3). The integrated approach was subsequently used for two different sample sets, one including all 64 samples and one including a subset of 10 individuals (5 males and 5 females randomly chosen) from each population, using the chosen parameter settings for *m*, *M*, and *n*, and the *E. lucius* reference genome (published by Rondeau et al., 2014) was used for alignment of the sequences. This created a catalog containing 128,884 loci. Following catalog construction, the Populations software in Stacks was used to identify the loci in the catalog that were present in at least 80% of the samples within each population, and that were shared by all three populations. This procedure was run separately for the full sample size (*N* = 64) and the subsetted sample size (*N* = 30) respectively (henceforth referred to as “full SNP dataset” and “subset SNP dataset”). For this, a minimum read depth of 10, minimum minor allele frequency of 0.05, and a maximum observed heterozygosity of 0.70 was used. To avoid linkage between markers, only one random SNP per locus was kept. After this relatively strict filtering with Populations, the final dataset consisted of 1580 and 1670 biallelic SNPs for the full as subset SNP datasets respectively. Both datasets were separately used in the subsequent analyses of population structure, genetic diversity and migration, and the dataset for the full SNP dataset was additionally used for outlier analyses. All the analyses were also performed when excluding individuals with >10% missing data (one male from Lervik). The exclusion resulted in only minor changes in the specific values of the estimates but did not qualitatively change the results; and because the results were robust we chose not to exclude the individual from the analyses.

### Analysis of Genetic Diversity and Population Structure

To test for scoring errors in microsatellites due to stuttering and allelic dropouts MICRO-CHECKER v2.2.3 (van Oosterhout et al., 2004) was used with 95% confidence intervals and 1000 permutations, and the test did not find evidence of scoring errors due to stuttering or allelic dropouts. To estimate the frequency of null alleles the excluding null allele (ENA) algorithm implemented in FreeNA (Chapuis and Estoup, 2006) was used with 1000 permutations, and the impact of null alleles on the analysis of population differentiation was evaluated by calculating fixation index (*F*_ST_) with and without excluding null alleles. The frequency of null alleles for each loci in each population was relatively low (<10%; Supplementary Table S1), with the exception of one locus (Elu-6) in one population (Harfjärden) that had a higher frequency (17%). The presence of null alleles in the one locus in a single population did not qualitatively affect *F*_ST_ values per locus over populations or pairwise *F*_ST_ values between populations over loci (Supplementary Table S2). Therefore, we continued with the uncorrected allele frequencies for subsequent analyses. The microsatellite dataset was, as the SNP dataset analyzed both in its entirety and when subsetting the same 10 individuals from each populations as were used for the subset SNP dataset (henceforth referred to as “full microsatellite dataset” and “subset microsatellite dataset”).

For each of the two microsatellite datasets, mean number of alleles, observed heterozygosity (*H*_O_), expected heterozygosity (*H*_E_), and departures from Hardy-Weinberg equilibrium (HWE) were estimated in Arlequin v3.5 (Excoffier and Lischer, 2010), allelic richness (*Ar*) and the fixation index *Fis* were calculated with the software FSTAT (version 2.9.4) (Goudet, 1995), and number of private alleles was calculated in GENALEX v6.5 (Peakall and Smouse, 2006). For both of the SNP datasets total number of alleles, number of variant loci, number of private alleles, observed heterozygosity (*H*_O_), expected heterozygosity (*H*_E_), and fixation index (*Fis*) were estimated using the Populations software in the Stacks pipeline (for details about parameter settings see section Quality Control and Genotyping above).

Multiple approaches that use different algorithms were utilized to investigate the genetic structure and dynamics among the three populations. The purpose of using multiple approaches was to be able to compare the results from the different analyses to obtain a more comprehensive view of the structure detected by the different marker types and sample sizes. Pairwise population differentiation was assessed with the fixation index *F*_ST_ (Weir and Cockerham, 1984) in GENALEX (Peakall and Smouse, 2006) for the two microsatellite datasets (using 9,999 permutations), and were obtained from the Populations software in Stacks for the two SNP datasets. To determine the most likely number of genetic clusters (*K*) STRUCTURE v2.3.4 (Pritchard et al., 2000) was used for each of the two microsatellite datasets, and the corresponding software for large SNP datasets, fastSTRUCTURE v1.0 (Raj et al., 2014), was used for each of the two SNP datasets. Because the proportion of loci identified as putatively under selection was low (∼1% for the full SNP dataset, see section Identification of Loci Putatively Under Selection in Results), the effect of non-neutral loci should not have a large effect on the results, and we therefore chose to use all SNPs in the population structure analyses. Both STRUCTURE and fastSTRUCTURE use a Bayesian approach to assign individuals to genetic clusters. STRUCTURE was run with *a priori* population information, admixture model, correlated allele frequencies and the default settings according to Pritchard et al. (2000) using a burn-in period of 100,000 generations followed by 200,000 sampling generations. The procedure was iterated 10 times for each *K*, where *K* ranged from one to six. The most likely number of genetic clusters was determined with the method by Evanno et al. (2005) implemented in the software STRUCTURE HARVESTER v0.6.94 (Earl and vonHoldt, 2012), and the CLUMPAK Server was used to combine and visualize the STRUCTURE results (Kopelman et al., 2015). fastSTRUCTURE was run separately for the two SNP datasets, for each *K* from one to 10 with the provided script structure.py, using a simple prior. The most likely number of genetic clusters was determined for both the full SNP dataset and the subset SNP dataset with the script chooseK.py, and distruct plots for the chosen values of *K* were created using the script distruct. py (Raj et al., 2014). The patterns of genetic differences among the three populations based on all four datasets were visualized with Canonical Analysis of Principal coordinates (CAP) (Anderson and Willis, 2003) in PRIMER7, which is a constrained ordination method that projects the multivariate objects to the axes that maxime the variation between predefined groups (sampling locations in the present study). Prior to the analyses, a diagnostic analysis for each dataset was performed with CAP to determine the number of axes (*m*) to use. The *m* values suggested by the software (full SNP: 37, subset SNP: 9, full microsatellite: 4, subset microsatellite: 3) were chosen and used in the further CAP analyses. The null hypothesis of no difference between the centroids of the populations was tested with CAP using 9,999 permutations (Anderson and Willis, 2003; Anderson et al., 2008). Cross-validation was performed using the leave-one-out approach, which calculates the percentage of samples correctly assigned to their predefined groups.

In addition, a Principal Component Analysis (PCA) based on pairwise genetic similarity (proportion of alleles shared) between individuals and a K-means clustering analysis in Discriminant Analysis of Principal Components (DAPC) (Jombart et al., 2010) were performed for each of the four datasets. These approaches were included as being – unlike STRUCTURE, fastSTRUCTURE and CAP analysis – model-free with no pre-assumptions about the populations (including being in HWE). PCA was visualized with the prcomp function in RStudio 2 v1.1.383 (RStudio Team, 2015), with R v3.2.2 (R Core Team, 2012), and the adegenet package (v. 2.1.1) in R was used for DAPC. To avoid overfitting, the number of principal components (PCs) that minimized the mean square error was determined for each of the four dataset and used in the DAPC analyses. Cross-validation for DAPC was carried out for each of the four datasets using 100 repetitions for each PC retention (and 90% as training set and 10% as validation set), and resulted in the following numbers of PC retention: 5 PC for full microsatellite, 8 PCs for subset microsatellite, 20 PC for full SNP dataset, and 18 PC for subset SNP.

### Estimation of Migration

Recent migration rates (m) among the populations were estimated from all four datasets (full and subset for both microsatellites and SNPs) with a Bayesian Markov Chain Monte Carlo (MCMC) resampling method in BAYESASS v3.0.4 (Wilson and Rannala, 2003), which estimates the proportion of migrants per generation within the last few generations. Optimization of parameters was done through preliminary analyses before the real tests were performed. Mixing rates for allele frequencies, inbreeding coefficients and migration rates were adjusted to achieve the suggested range of 20–40% (Wilson and Rannala, 2003) for convergence. The analysis was run with a burn−in of 1 million iterations, followed by 10 million iterations with sampling at every 1,000 steps. Convergence was confirmed by plotting the cumulative log likelihoods of the iterations using the program TRACER 1.7 (Rambaut et al., 2018), and four runs were performed using different random seed numbers to ensure consistency. Each dataset was also analyzed with GeneClass2 (Piry et al., 2004) to identify first generation migrants using the Bayesian criterion of Rannala and Mountain (1997). The likelihood computation was based on Lhome/Lmax with a Monte-Carlo resampling algorithm (Paetkau et al., 2004) with 1,000 simulations and α = 0.001.

### Identification of Loci Putatively Under Selection

Neutrality of the microsatellite markers was tested using the default settings in BayeScan, and the results suggested that none of the microsatellite were under selection (*q*-values 0.53–0.89, Supplementary Table S3). For the tests of outlier loci, only the full SNP dataset was therefore used. For this, several approaches were utilized to find loci putatively under selection, to be able to compare the results from the different approaches, and to include testing for environmental correlations. Because different approaches use different algorithms and assumptions, and have different types of limitations (de Villemereuil et al., 2014), the SNPs identified as being under selection might differ between the methods. Therefore, to be confident that the outliers are likely to be true positives, they should preferably be identified by multiple approaches (de Villemereuil et al., 2014).

First, we used two *F*_ST_-based approaches to test for SNPs under selection: a Bayesian approach implemented in BayeScan v.2.1 (Foll and Gaggiotti, 2008), using 100 as prior odds for neutral model and otherwise the default settings, and a coalescent simulation approach (Fdist) in Arlequin v3.5 (Excoffier and Lischer, 2010) with 100,000 simulations, 100 demes, minimum heterozygosity of 0.05, maximum heterozygosity of 0.70, and a finite island model to find loci under selection.

Second, to test for correlation between selection and environmental variables we used BayeScEnv and latent factor mixed model (LFMM). BayeScEnv 1.1 aims to differentiate between signals of selection and signals of purely demographic processes on allele frequencies (de Villemereuil and Gaggiotti, 2015), by identifying loci for which genetic differentiation is associated with a chosen environmental factor. Convergence of the MCMC chains was confirmed by plotting the log-likelihood trace in CODA package in R (Plummer et al., 2006). For LFMM, missing genotypes were imputed using Beagle 5.0 (Browning et al., 2018), and the results from the PCA, and a test of cross-entropy (for *K* 1–10) performed with the LEA package in R (Frichot and François, 2015) were used to determine the most likely number of *K* to use, which suggested a value of three. The LFMM analyses were performed with the LFMM package in R (Caye and François, 2018), and were run separately for two different environmental covariables (salinity and temperature). To estimate the values for the environmental variables, we used the median salinity in the spawning ground during spawning (Harfjärden 0 psu, Lervik 3 psu, and Okne 0 psu; Sunde et al., 2018a, Jonas Nilsson unpublished data), and temperature at initiation of spawning (Harfjärden 4.3°C, Lervik 9.4°C; Sunde et al., 2019, and Okne 6.0°C, Jonas Nilsson unpublished data). The environmental values were normalized against “normal” values for the environmental variables (to a standard deviation of 1; by subtracting the normal value from the observed value, and dividing by the standard deviation of all observed values). For this 0 (psu) was used as the normal value for salinity, because pike has a freshwater origin (Raat, 1988; Craig, 1996), and the mean temperature of the three populations (6.6°C) as normal value for temperature at initiation of spawning.

FDR correction of *P*-values, to adjust for multiple testing, was performed using the method by Benjamini and Hochberg (1995). Significance threshold was set to FDR corrected *P*-values (*q*-values) = 0.05 for all outlier analyses. To identify candidate genes, all loci suggested as putatively being under selection or associated with the environmental factors in the outlier analyses were annotated using the reference genome (published by Rondeau et al., 2014).

### Literature Search and Synthesis of Previous Studies That Have Used Both Microsatellites and RADseq Markers to Investigate Population Genetic Structure

To identify previous studies that have used both microsatellites and RADseq markers for studing population genetic structure we conducted a topic search on 25 June, 2019, in ISI Web of Science (Data base: core collection; Time span: 1900-2019) using the search strings listed in Figure 1. For the identified studies we extracted and synthsized information on species, sample sizes (number of populations and individuals), number of markers used, and summarized the overall findings and conclusions (Table 1). Lastly, we combine the results of our own analysis of pike with the findings of seven previous studies to evaluate whether relative performance of microsatellites and RADseq markers are general or context specific.

**TABLE 1:** Publications in which both RADseq and microsatellite data were utilized to investigate population genetic structure of various organisms (survey date: 25-06-2019).

			**Microsatellites**					**RADseq**					
**References**	**Species**	**Organism**	***N***	***N* pop**	***N* tot**	***N* ind**	**H_O_/H_E_**	***N***	***N* pop**	***N* tot**	***N* ind**	**H_O_/H_E_**	**Findings**
Bradbury et al., 2015	Atlantic salmon *Salmo salar*	Fish	15	32	2439	51–111	0.70–0.88 (*H*_O_)	8495	16	313	13–20	0.230 (mean *H*_O_)	Similar trends with both markers. RADseq better at detecting introgression. No significant difference in the observed heterozygosity.
Jeffries et al., 2016	Crucian carp *Carassius carassius*	Fish	13	49	848	4–37	0.25 (mean *H*_O_)	13189	18	149	8–10	0.013 (mean *H*_O_)	Similar trends with both markers. RADseq better at detecting fine-scaled genetic structure despite smaller sample sizes.
Hodel et al., 2017	Red mangroves *Rhizophora mangle*	Tree	8	12	96	8	0.431 (*H*_O_) 0.388 (*H*_E_)	239–25,198	12	96	8	0.356–0.477 (*H*_O_) 0.300–0.340 (*H*_E_)	RADseq provided increased phylogeographic resolution.
Morgan et al., 2017	Round whitefish *Prosopium cylindraceum*	Fish	9	14	390	8–60	*NA*	8835	14	190	3–32	*NA*	Similar trends with both markers. RADseq offered enhanced resolution of genetic structure.
Guzinski et al., 2018	Pacific kelp *Undaria pinnatifida*	Brown algae	10	33	1111	*NA*	*0.108–0.471* (*H*_E_)	10,615	33	706	2–24	0.037–0.151 (*H*_E_)	Similar trends with both markers. RADseq better at identifying fine-scaled genetic structure.
Lemopoulos et al., 2019	Brown trout *Salmo trutta*	Fish	16	4	120	30	0.48–0.66 (*H*_E_)	4876	4	75	9–29	0.09–0.14 (*H*_E_)	Similar trends with both markers. RADseq better at estimating individual−level multilocus heterozygosity.
Bohling et al., 2019	Bull trout *Salvelinus confluentus*	Fish	16	24	322	*NA*	*NA*	79,952	24	344	8–16	*NA*	Similar trends with both markers. RADseq provided increased phylogeographic resolution.
This study	Northern pike *Esox lucius*	Fish	10	3	64	21–22	0.400–0.565 (*H*_O_) 0.452–0.585 (*H*_E_)	1580	3	64	21–22	0.208–0.289 (*H*_O_) 0.207–0.284 (*H*_E_)	Similar trends with both markers. RADseq provided more conclusive results of higher resolution of genetic structure.

**N, Number of markers; N pop, Number of populations; N tot, Total number of individuals in the study; N ind, Number of individuals per population; H_O_/H_E_, Estimated heterozygosity (H_O_, observed heterozygosity; H_E_, expected heterozygosity).**

## Results

### Genetic Diversity and Population Structure

For the 10 microsatellite loci, three to 28 alleles were found across populations with a mean of 3.4–4.8 alleles across loci per population (4.0–4.8 for full dataset, and 3.4–4.3 for subset dataset, see Table 2 and Supplementary Table S4 for details). According to the microsatellite datasets, heterozygosity levels were relatively high (full microsatellite dataset *H*_O_ = 0.40–0.57, subset microsatellite dataset *H_O_* = 0.43–0.64), and all three populations were in agreement with Hardy-Weinberg expectation at the majority of the loci (with the exception of one or two loci per population; see Supplementary Table S4).

**TABLE 2:** Molecular diversity indices across loci for each population for the full datasets: microsatellites (“Microsatellite”), RADseq SNPs (“RADseq”), and for the subset datasets (10 individuals per population): microsatellites (“Microsatellite subset”) and RADseq SNPs (“RADseq subset”).

	**Microsatellite**								**RADseq**						
**Population**	***N***	***Na***	**Mean *Na* (SD)**	**Ar**	**PA**	***H*_O_ (SD)**	***H*_E_ (SD)**	***Fis***	***N***	**Variant sites**	**PA**	**Polymorphic loci**	***H*_O_ (SE)**	***H*_E_ (SE)**	***Fis* (SE)**
Lervik	22	45	4.50 (2.72)	4.78	10	0.565 (0.208)*	0.566 (0.153)	0.003	22	1580	50	1413	0.2736 (0.0047)	0.2764 (0.0042)	0.0292 (0.0282)
Okne	21	48	4.80 (1.93)	4.54	16	0.556 (0.175)*	0.585 (0.190)	0.052	21	1580	36	1416	0.2886 (0.0048)	0.2842 (0.0042)	0.0085 (0.0263)
Harfjärden	22	42	4.10 (3.93)	4.47	14	0.400 (0.122)*	0.452 (0.199)	0.118	22	1580	60	1193	0.2078 (0.0047)	0.2070 (0.0044)	0.0134 (0.0276)

	**Microsatellite subset**								**RADseq subset**						
**Population**	***N***	***Na***	**Mean *Na* (SD)**	**Ar**	**PA**	***H*_O_ (SD)**	***H*_E_ (SD)**	***Fis***	***N***	**Variant sites**	**PA**	**Polymorphic loci**	***H*_O_ (SE)**	***H*_E_ (SE)**	***Fis* (SE)**

Lervik	10	35	3.50 (1.35)	3.82	5	0.637 (0.226)*	0.576 (0.153)	−0.085	10	1670	88	1407	0.2641 (0.0050)	0.2670 (0.0042)	0.0469 (0.0173)
Okne	10	39	3.90 (1.45)	4.23	12	0.500 (0.156)*	0608 (0.190)	0.131	10	1670	85	1376	0.2787 (0.0051)	0.2667 (0.0042)	0.0051 (0.0143)
Harfjärden	10	34	3.40 (2.76)	3.52	12	0.430 (0.142)*	0.474 (0.213)	0.098	10	1670	87	1084	0.1987 (0.0051)	0.1947 (0.0044)	0.0213 (0.0139)

N, number of samples; Na, total number of alleles; Mean Na, number of mean alleles over loci; Ar, allelic richness; PA, number of private alleles; H_O_, observed heterozygosity; H_E_, expected heterozygosity; Fis, fixation index; SE, standard error; SD, standard deviation. Significant deviation from HWE (*P < 0.05).

Estimates of observed heterozygosity were lower for both RADseq SNP datasets (full SNP dataset *H*_O_ = 0.21–0.29, subset SNP dataset *H_O_* = 0.20–0.28) than those obtained for the microsatellite datasets. The majority of the loci in the SNP datasets were in HWE (50–64 loci for each population were not, *P* < 0.05), and yet *Fis* was close to zero for all populations (Table 2). In addition, for all populations, the *Fis* distributions for both SNP datasets were unimodal and peaking at zero (Supplementary Figure S4), which suggests that all three populations met the expectations of HWE, and the majority of the loci was not affected by null alleles (Ravinet et al., 2016).

**FIGURE 3:**
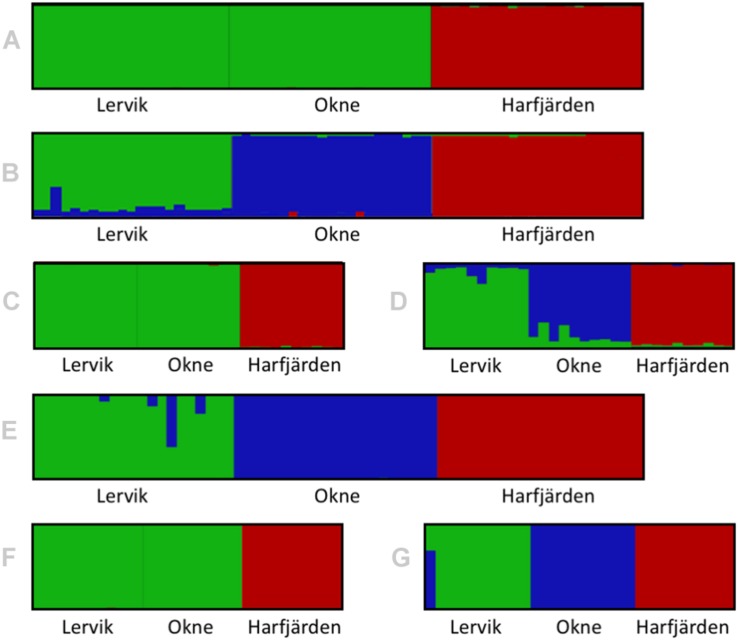
Structure/distruct-plots for the most likely number of genetic clusters (*K*) in the sampled individuals for full (*N* = 64) and subset (*N* = 30) datasets for both microsatellites analyzed with STRUCTURE **(A–D)**, and RADseq SNPs analyzed with fastSTRUCTURE **(E–G)**. Plots show the results for **(A)** full microsatellite dataset for *K* = 2, **(B)** full microsatellite dataset for *K* = 3, **(C)** subset microsatellite dataset for *K* = 2, **(D)** subset microsatellite dataset for *K* = 3, **(E)** full RADseq SNP dataset for *K* = 3, **(F)** subset RADseq SNP dataset for *K* = 2, and **(G)** subset RADseq SNP dataset for *K* = 3.

**FIGURE 4:**
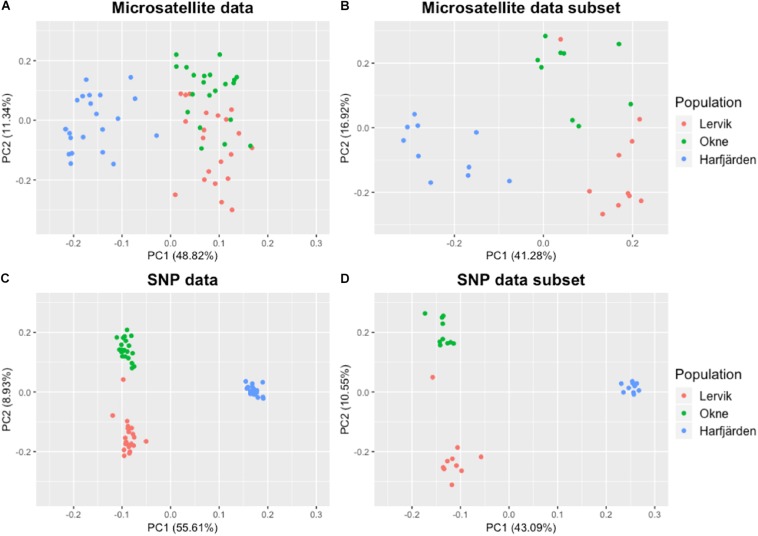
PCA plots based on pairwise similarity (proportion of alleles shared) among individuals. Plots are based on **(A)** full microsatellite dataset (10 loci, *N* = 64), **(B)** subset microsatellite dataset (10 loci, *N* = 30), **(C)** full RADseq SNP dataset (1580 biallelic SNPs, *N* = 64), and **(D)** subset RADseq SNP dataset (1670 biallelic SNPs, *N* = 30).

Genetic differentiation estimates (*F*_ST_) based on all four datasets (full and subset microsatellite, and full and subset RADseq) suggested differentiation among all three populations (*F*_ST_ = 0.059–0.172, *P* < 0.01 for all pairwise comparisons, Table 3). The results from the fastSTRUCTURE analysis of the full SNP dataset suggested that the most likely number of genetic clusters (*K*) was three (for both the model complexity that maximizes marginal likelihood and for model used to explain structure in the data), and revealed that the three clusters were mainly population-specific (Figure 3E). For the subset SNP dataset, fastSTRUCTURE suggested most likely number of *K* as either two (for model complexity that maximizes marginal likelihood) (Figure 3F) or three (for model used to explain structure in the data) (Figure 3G), and the PCA were in support of the populations belonging to three mainly population-specific clusters (Figure 4D). When running STRUCTURE HARVESTER on the output from the STRUCTURE analyses of the two microsatellite datasets (full and subset dataset), it suggested that the most likely number of genetic clusters were two for both datasets. However, the Evanno method can be unreliable for small values of *K*, and based on the LnPk and deltaK plots (Supplementary Figure S5) three clusters could also be considered. Both microsatellite datasets revealed similar patterns, and showed that for *K* = 2 the two geographically most adjacent populations (Lervik and Okne) were assigned to the same genetic cluster (Figures 3A,C), whereas *K* = 3 indicated that the populations mainly belong to population-specific clusters (Figures 3B,D). The results from the PCAs were largely consistent with those from the STRUCTURE and fastSTRUCTURE analyses. For both SNP datasets (full and subset), the PCAs revealed three distinct clusters, one for each of the populations (Figures 4C,D). For the microsatellites, the full dataset suggested two clusters, one for the Harfjärden population and one for Lervik and Okne combined (Figure 4A), and the subset dataset could be interpreted as either two or three clusters (Figure 4B).

**TABLE 3:** Pairwise population differentiation estimates.

**Comparison**	**Microsatellites**	**Microsatellites**	**RADseq**	**RADseq**
		**subset**		**subset**
Lervik – Okne	0.068	0.071	0.059	0.069
Okne – Harfjärden	0.156	0.148	0.156	0.136
Harfjärden – Lervik	0.169	0.172	0.162	0.137

**F_ST_ values estimated based on microsatellite data (“Microsatellites”), and RADseq SNP data for all samples (“RADseq”) and a subset of 10 individuals from each population (“RADseq subset”). P ≤ 0.01 for all comparisons.**

Because additional separation among clusters (especially between Lervik and Okne) could be hidden in another dimension of the PCAs we also utilized two ordination methods, one with *a priori* population information (CAP, Supplementary Figure S6), and one without (DAPC, Supplementary Figures S7, S8). The CAP analyses showed that the proportion of variation explained with the suggested *m*-values were 83 and 61% for the full and subset RADseq SNP datasets respectively, and 69 and 63% for the full and subset microsatellite datasets respectively. Trace statistics (*tr*: the sum of the squared canonical correlations) supported a significant differentiation between the centroids of the populations for all datasets (microsatellite: full, *tr* = 1.43; subset, *tr* = 1.43; SNP: full, *tr* = 1.98, subset *tr* = 1.96; *P* < 0.0001 for all tests). The cross-validation with the leave-one-out method showed that the assignment success was 100% (64/64) with the full RADseq SNP dataset, and 96.7% (29/30) for the subset SNP dataset – which mis-assigned LF3 (the potential migrant identified by GeneClass, see the Results section Migration below) to Okne. For the microsatellite datasets, assignment success was also high: 93.8% (60/64) and 93.3% (28/30) for the full and subset datasets respectively. For the full dataset three individuals from Lervik were assigned to Okne, and for the subset dataset one individual from Lervik was assigned to Okne, and one individual from Okne was assigned to Lervik.

The K-means clustering analyses with DAPC clearly suggested three clusters for both SNP datasets (Supplementary Figures S7C,D). The most likely number of clusters for the microsatellite datasets were not as clear as for the SNP datasets. The results for both microsatellite datasets indicated that three clusters could be possible. However, the elbow in the plot for the full microsatellite dataset was not really distinct, and the lowest Bayesian Information Criterion (BIC) was obtained for *K* = 5 (Supplementary Figure S7A); and for the subset microsatellite dataset the BIC continuously decreased with increasing *K* and did not stabilize (Supplementary Figure S7B). The assignment test revealed that the assignment success was 98.4% (63/64) and 96.7% (29/30) for the full and subset SNP datasets respectively, and 89.1% (57/64) and 90.0% (27/30) for the full and subset microsatellite datasets respectively (Supplementary Figure S8).

### Migration

TRACER showed that migration rates converged on similar estimates (before 500,000 generations) for all BAYESASS runs. Gene flow estimates for all population pairs were low for both of the full datasets (microsatellite 1.5–4.0%, SNP 1.3–6.1%, Supplementary Figures S9A,C), and for the subset SNP dataset (2.5–5.2%; Supplementary Figure S9D). The estimates for the subset microsatellite dataset were also low for the mainland – island population pairs (2.8–4.3%), but were higher between the two mainland populations (9.8–26.9%) (Supplementary Figure S9B). The results from the GeneClass2 analyses also suggested low levels of gene flow, and only identified a few (one to two) first generation migrants (Supplementary Tables S5, S6). The full microsatellite dataset indicated two first generation migrants, one individual in the Okne population that immigrated from Lervik, and vice versa, and revealed no migration events between Harfjärden and the other two populations (Supplementary Table S5). The subset microsatellite dataset and both SNP datasets suggested only one first generation migrant each (Supplementary Tables S5, S6), which was the same individual in Lervik immigrating from Okne as identified by the full microsatellite dataset.

### Identification of Loci Putatively Under Selection

**FIGURE 5:**
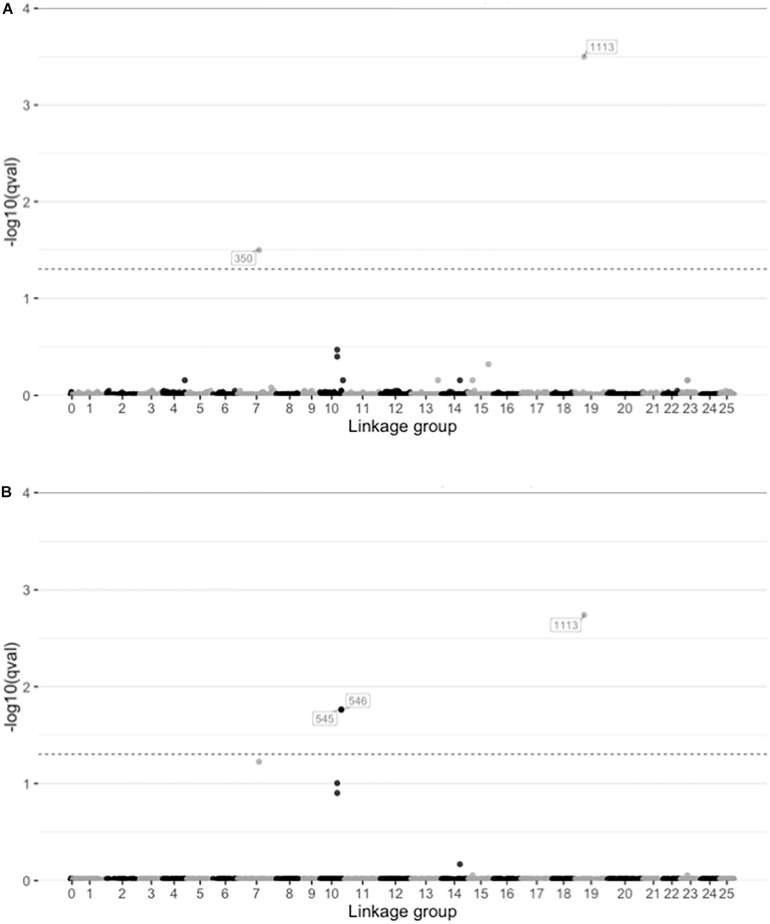
Plots showing the results from latent factor mixed model (LFMM) analysis of SNPs correlated with environmental variables. Plots show the -log10 *q*-values for each SNP distributed along the linkage groups (linkage group 0 indicate unplaced scaffolds) for two environmental variables **(A)** salinity in the spawning ground, and **(B)** temperature at initiation of spawning. The dashed lines indicate significance threshold (*q* = 0.05).

Among the several approaches utilized, only the LFMM approach identified any candidate SNPs putatively under selection. The LFMM analyses identified 2 and 3 SNPs correlated with salinity and temperature respectively (*q* < 0.05; Figure 5 and Supplementary Table S7). No locus-specific effects were found with either BayeScan (*q*-values 0.81–0.99) or Fdist (*q*-values 0.23–1.00), and BayeScEnv did not find any signals of selection associated with either salinity (*q*-values 0.22–0.96; Supplementary Figure S10A) or temperature (*q*-values 0.28–0.98; Supplementary Figure S10B).

### Review and Synthesis of Previous Studies

The literature search identified seven previous studies that have used both microsatellites and RADseq markers for studing population genetic structure (Table 1). Most of the studies used higher numbers of populations and/or individuals for microsatellites than for RADseq, and only two of the seven studies included the exact same set of populations and individuals for both marker types. As for study organisms, two of the seven studies used plant species (one species of brown algae and one tree species), and the remaining five studies used different species of fish; no other animal group was represented.

Five of the seven studies reported within population heterozygosity (*H*_O_ and/or *H*_E_), and for all of these five studies, the estimates of heterozygosity were equal or lower for RADseq compared to microsatellites. Three of the five showed lower values for RADseq compared to microsatellites, and the other two studies found no significant differences (Table 1). This is consistent with the results in the present study, that also obtained lower estimates of heterozygosity for RADseq than for microsatellites, and reflects a methodological/technical difference between the marker types.

Regarding genetic structure, the findings from the studies revealed that both marker types produced comparable results for highly divergent populations, but that RADseq was more successful than microsatellites in revealing fine-scaled population structure in studies where > 400 loci were utilized (Table 1). This is in line with the findings in the present study where we found that both marker types were able to detect population structure, but that the full RADseq SNP dataset (*N* = 64) provided clearer results and a more conclusive picture of the population structure.

## Discussion

### Comparison of Heterozygosity Estimates Based on Microsatellites and RADseq SNPs

When analyzing the RADseq SNP data, estimates of heterozygosity were similar for the full sample size and for the subset of individuals (*H*_O_ 0.21–0.29 for the full SNP dataset and 0.20–0.28 for the subset; Table 2). For the full and subset microsatellite datasets, the heterozygosity estimates differed somewhat more (*H*_O_ 0.40–0.57 for the full microsatellite dataset and 0.43–0.64 for the subset; Table 2). There was also a relatively large difference in estimates obtained with the two different marker types, and values for RADseq were consistently lower than for microsatellites. Discrepancies in heterozygosity levels between the marker types have also been observed previously (Table 1), and it is important to be aware of such differences when interpreting heterozygosity levels. The observed difference is likely influenced by technical and methodological differences rather than biological differences. Because of the differences in the inherent properties, comparisons of heterozygosity estimates obtained with the different markers should not be on the specific values, but on the ranking of populations or the relative differences among populations.

Overall genetic diversity reflects past events, and the level of functional genetic diversity within populations can impact the ability to cope with changing and novel environmental conditions (Hughes et al., 2008; Forsman, 2014; Charlesworth and Charlesworth, 2017; Yıldırım et al., 2018b). Given the inherent differences between marker types, assessments of vulnerability of populations based on estimates of genetic diversity must be method-specific. In conservation contexts, a way of assessing potential negative manifestations of inbreeding is by investigating whether individual heterozygosity is associated with performance. The presence of a heterozygosity-fitness correlation, such that low heterozygosity is associated with decreased performance (HFC; David, 1998), would indicate inbreeding depression. However, HFCs are often weak, and require that the diversity estimates reflect genome-wide diversity (Chapman et al., 2009). This in turn requires that a large number of markers are used, regardless of marker type, to avoid false acceptance of the null hypothesis that inbreeding has no negative effects.

### Comparison of Estimates of Population Structure Based on Microsatellites and RADseq SNPs

When analyzing population structure, the different approaches yielded somewhat mixed results. Based on *F*_ST_ values, the findings were consistent, and all four datasets (full and subset for both microsatellite and SNP datasets) revealed significant differentiation among all three populations. Both marker types also revealed similar patterns of the degree of pairwise population differentiation (Table 3). Consistent with these findings, both fastSTRUCTURE and PCA (for the full SNP dataset) also suggested the presence of three mainly population-specific genetic clusters (Figures 3E, 4C). The results for the subset SNP dataset were somewhat less conclusive but suggested similar trends to those of the full sample SNP dataset; for fastSTRUCTURE the most likely number of genetic clusters was suggested to be either two or three, and in combination with the PCA it was evident that the individuals were separated in three mainly population specific genetic clusters (Figures 3G, 4D). Like the results for the subset SNP dataset, the results for the microsatellites were somewhat inconclusive and both the full and the subset dataset indicated that either two or three clusters could be possible (Figures 3A,D, 4A,B). Both the STRUCTURE analyses and the PCAs suggested that the populations most likely belonged to only two genetic clusters, and that Harfjärden belonged to one population-specific genetic cluster, whilst Lervik and Okne were both assigned to the same genetic cluster. However, the results also indicated that there could be three clusters, and when using *K* = 3 STRUCTURE assigned the populations to three mainly population-specific clusters also for both microsatellite datasets. For the PCA analyses, even though it is possible that the overlapping clusters found with the microsatellite datasets are separated in another dimension, the majority of the variation seem to be explained by the separation between the mainland populations (Lervik and Okne) and the island population (Harfjärden) (>40% on first PC for all datasets). To evaluate whether separation in other dimensions affected the estimated population structure, we used DAPC, which effectively collapses the dimensions of the PCA into a single measure of separation. The results from DAPC revealed that both SNP datasets clearly suggested three clusters, whilst the results for the microsatellites were not as conclusive, and did not clearly suggest any specific value of *K* (Supplementary Figure S7). The CAP analysis on the other hand indicated distinctiveness of the three populations for all four datasets (Supplementary Figure S6). This discrepancy between the findings in the DAPC and the CAP analyses is likely explained by that *a priori* population information is used in the CAP analysis, but not in DAPC. The assignment tests revealed that all of the identified mis-classifications had occurred between the more closely related populations (Lervik and Okne). In CAP cross-validation for the full SNP dataset, all individuals were assigned to their respective population. For the subset SNP dataset one individual was mis-classified, which was most likely a first generation migrant (suggested by GeneClass). The cross-validation test for the microsatellite datasets mis-classified three and two individuals for the full and subset datasets respectively, and these were not the same individuals as those identified as migrants using GeneClass.

The results of the migration analyses (with BAYESASS and GeneClass) for all four datasets indicated low levels of gene flow for all population pairs (1.3–6.1%, 1–2 first generation migrants; Supplementary Figure S9 and Supplementary Tables S5, S6), except for the GeneClass results for gene flow between Lervik and Okne based on the subset microsatellite dataset (9.8–26.9%, Supplementary Table S6). Both marker types thus seemed to be able to detect migrants, and yielded comparable results for both of the full datasets and the subset SNP dataset.

A likely explanation for the findings regarding the estimated number of populations is that Lervik and Okne are actually two separated populations. This is supported by the results from a more comprehensive microsatellite study of pike in the Kalmar Sound by Nordahl et al. (2019), in which a total of 457 pike from 13 populations (including the three populations included in the present study, but not the same set of individuals) were genotyped for the same set of microsatellite markers as used in the present study. In that study larger sample sizes (36–69 individuals per population) were used, and the results revealed that the three populations were genetically differentiated, and that Lervik and Okne did mainly belong to separate population-specific genetic clusters. The findings reported in the previous studies of plants and animals identified in our literature survey (Table 1) indicate that RADseq are more successful than microsatellites in revealing fine-scaled genetic structure when > 400 SNPs are used, even when fewer individuals have been sampled. This may partly reflect that only few microsatellite loci were utilized in all the studies included in the synthesis. More recent studies utilizing next generation sequencing to recover hundreds of microsatellite markers (e.g., Bradbury et al., 2018) might provide more comparable estimates of genome-wide diversity and better resolution of genetic structure. The relative performance of the two markers should therefore be further investigated when utilizing higher numbers of microsatellites as well.

### Identification of Loci Putatively Under Selection

We have clear indications from previous studies based on different approaches (common garden and translocation experiments) that the three populations included in the present study are adaptively differentiated. The local adaptations have resulted from environmental differences among the spawning locations, and can for example be attributed to differences in the salinity and temperature regimes (Sunde et al., 2018a, 2019), and the amount of suspended materials (Berggren et al., 2016). Because of this prior knowledge about local adaptations in the populations, testing for genetic signatures of selection was of particular interest to identify candidate genes responsible for the observed phenotypic differences. It also enabled us to test the capability of RADseq SNPs to detect signatures of selection associated with the environmental factors already implicated as imposing selective pressures.

Despite the already existing evidence of phenotypic local adaptation in the populations, the two analyses of locus-specific effects (Bayescan and Fdist) did not identify any SNPs under selection. This finding was somewhat surprising, because adaptive differentiation between the populations should be reflected in the genome. However, the LFMM analyses did identify signatures of selection, when salinity and temperature were introduced as environmental factors (Figure 5 and Supplementary Table S7).

Of the SNPs identified as outliers by the LFMM analyses (two for salinity and three for temperature, after correction for multiple comparisons), one SNP was associated with both of the environmental variables, which indicates that it might not be directly associated with a single environmental variable, but rather stress in general. The annotation revealed that of the four SNPs identified, three were located within known genes (Supplementary Table S7). It is possible that the last SNP might also be located in a gene that is not yet identified in the pike genome, but it is also possible that these are non-coding loci linked to regions under selection. However, LFMM has a tendency to produce high false positive rates for low dispersal populations (Forester et al., 2016), so caution should be taken before drawing firm conclusions about which genes are responsible for adaptation in such cases. To get a more comprehensive picture of the selective forces acting on this species, future studies should include a higher number of populations to achieve higher statistical power in the outlier analyses. Genome scans with higher density could thus potentially reveal some additional candidate loci under strong or moderate selection that were not represented in the present study.

Conclusions about the importance of different environmental factors should also be done cautiously when based on results from outlier analyses alone, as responses to selection are further complicated by developmental plasticity, phenotypic flexibility and crossing norms of reaction affecting the phenotypic expression of genetic variation in the study populations, especially during early stages of the development (Berggren et al., 2016). For example, previous studies point to genotype by environment effects on hatching success for Okne and Lervik populations (Berggren et al., 2016; Sunde et al., 2019). There is also some evidence of genetic differentiation between and within the Lervik and Harfjärden populations in developmental plasticity of some early life history traits (Sunde et al., 2019). Therefore, care must be taken when inferring about the role of selection as a driver of population divergence from results of outlier analyses based on genetic marker data alone, as they may generate misleading conclusions.

## Conclusion

When comparing the relative performance of microsatellites and RADseq SNPs in the present study, both markers indicated genetic differentiation between the populations (*F*_ST_ values) for both the full and subset datasets. In addition, both marker types were able to detect genetic structuring for both datasets – though the full SNP dataset provided somewhat more conclusive results. The previous studies that have compared the two marker types, invariably report that RADseq SNPs outperform microsatellites in detecting fine-scaled structuring. However, the number of microsatellite loci that have been used in those studies have generally been low, and to evaluate the relative performance studies using higher numbers of microsatellites should also be investigated. Estimates of within population diversity (heterozygosity) based on RADseq SNPs were lower than those based on microsatellites, consistent with the findings in previous studies, and likely reflecting the high mutational rate of microsatellites. Both marker types generally indicated low levels of gene flow between all populations. Results from the outlier analyses suggested signs of selection associated with salinity and temperature, two environmental factors that have been implicated as drivers of local adaptation of these pike populations also in previous studies using different approaches.

## Data Availability Statement

The datasets from this article are publicly available. The sequence data (raw reads) are available in the NCBI Sequence Read Archive (BioProject: PRJNA586770, submission ID: SUB6466329; Sunde et al., 2020a), and the microsatellite data in the Dryad Digital Repository (Sunde et al., 2020b).

## Ethics Statement

The study was carried out in accordance with all relevant applicable national guidelines for the care and use of animals. The laboratory used was approved as research facility (Dnr 5.2.18-482/14), and the study (including capture and sampling procedures) was granted ethical approval (approval Dnr ID 83 and 39-10), by the Ethical Committee on Animal Experiments in Linköping, Swedish Board of Agriculture, Sweden.

## Author Contributions

JS and AF conceived the study. JS, AF, and PT conducted the sampling of fishes and collection of DNA samples. JS and YY performed the laboratory work, bioinformatical analyses, and drafted the first manuscript. JS, AF, and YY contributed to interpreting the results. All authors commented on and agreed to the final version of the manuscript.

## Conflict of Interest

The authors declare that the research was conducted in the absence of any commercial or financial relationships that could be construed as a potential conflict of interest.

## References

[B1] Anderson M. J. Gorley R. N. Clarke K. R. (2008). *PERMANOVA for PRIMER: Guide to Software and Statistical Methods.* Plymouth: PRIMER-E.

[B2] Anderson M. J. Willis T. J. (2003). Canonical analysis of principal coordinates: a useful method of constrained ordination for ecology. *Ecology* 84 511–525. 10.1890/0012-9658(2003)084[0511:CAOPCA]2.0.CO;2

[B3] Andrews K. R. Good J. M. Miller M. R. Luikart G. Hohenlohe P. A. (2016). Harnessing the power of RADseq for ecological and evolutionary genomics. *Nat. Rev. Genet.* 17 81–92. 10.1038/nrg.2015.28 26729255 PMC4823021

[B4] Baird N. A. Etter P. D. Atwood T. S. Currey M. C. Shiver A. L. Lewis Z. A. (2008). Rapid SNP discovery and genetic mapping using sequenced RAD markers. *PLoS One* 3:e3376. 10.1371/journal.pone.0003376 18852878 PMC2557064

[B5] Bekkevold D. Jacobsen L. Hemmer-Hansen J. Berg S. Skov C. (2015). From regionally predictable to locally complex population structure in a freshwater top predator: river systems are not always the unit of connectivity in Northern Pike *Esox lucius*. *Ecol. Freshw. Fish* 24 305–316. 10.1111/eff.12149

[B6] Benjamini Y. Hochberg Y. (1995). Controlling the false discovery rate: a practical and powerful approach to multiple testing. *J. R. Stat. Soc. Ser. B* 57 289–300. 10.1111/j.2517-6161.1995.tb02031.x

[B7] Berggren H. Nordahl O. Tibblin P. Larsson P. Forsman A. (2016). Testing for local adaptation to spawning habitat in sympatric subpopulations of pike by reciprocal translocation of embryos. *PLoS One* 11:e0154488. 10.1371/journal.pone.0154488 27139695 PMC4854435

[B8] Bohling J. Small M. Von Bargen J. Louden A. DeHaan P. (2019). Comparing inferences derived from microsatellite and RADseq datasets: a case study involving threatened bull trout. *Conserv. Genet.* 20 329–342. 10.1007/s10592-018-1134-z

[B9] Bolger A. M. Lohse M. Usadel B. (2014). Trimmomatic: a flexible trimmer for Illumina sequence data. *Bioinformatics* 30 2114–2120. 10.1093/bioinformatics/btu170 24695404 PMC4103590

[B10] Bradbury I. R. Hamilton L. C. Dempson B. Robertson M. J. Bourret V. Bernatchez L. (2015). Transatlantic secondary contact in Atlantic Salmon, comparing microsatellites, a single nucleotide polymorphism array and restriction-site associated DNA sequencing for the resolution of complex spatial structure. *Mol. Ecol.* 24 5130–5144. 10.1111/mec.13395 26407171

[B11] Bradbury I. R. Wringe B. F. Watson B. Paterson I. Horne J. Beiko R. (2018). Genotyping-by-sequencing of genome-wide microsatellite loci reveals fine-scale harvest composition in a coastal Atlantic salmon fishery. *Evol. Appl.* 11 918–930. 10.1111/eva.12606 29928300 PMC5999200

[B12] Brieuc M. S. O. Ono K. Drinan D. P. Naish K. A. (2015). Integration of Random Forest with population-based outlier analyses provides insight on the genomic basis and evolution of run timing in Chinook salmon (*Oncorhynchus tshawytscha*). *Mol. Ecol.* 24 2729–2746. 10.1111/mec.13211 25913096

[B13] Browning B. L. Zhou Y. Browning S. R. (2018). A one-penny imputed genome from next-generation reference panels. *Am. J. Hum. Genet.* 103 338–348. 10.1016/j.ajhg.2018.07.015 30100085 PMC6128308

[B14] Catchen J. Hohenlohe P. A. Bassham S. Amores A. Cresko W. A. (2013). Stacks: an analysis tool set for population genomics. *Mol. Ecol.* 22 3124–3140. 10.1111/mec.12354 23701397 PMC3936987

[B15] Catchen J. M. Amores A. Hohenlohe P. Cresko W. Postlethwait J. H. (2011). Stacks: building and genotyping loci de novo from short-read sequences. *G3* 1 171–182. 10.1534/g3.111.000240 22384329 PMC3276136

[B16] Caye K. François O. (2018). LFMM 2.0: latent factor models for confounder adjustment in genome and epigenome-wide association studies. *bioRxiv* [Preprint]. 10.1101/255893

[B17] Chapman J. R. Nakagawa S. Coltman D. W. Slate J. Sheldon B. C. (2009). A quantitative review of heterozygosity–fitness correlations in animal populations. *Mol. Ecol.* 18 2746–2765. 10.1111/j.1365-294X.2009.04247.x 19500255

[B18] Chapuis M.-P. Estoup A. (2006). Microsatellite null alleles and estimation of population differentiation. *Mol. Biol. Evol.* 24 621–631. 10.1093/molbev/msl191 17150975

[B19] Charlesworth B. Charlesworth D. (2017). Population genetics from 1966 to 2016. *Heredity* 118 2–9. 10.1038/hdy.2016.55 27460498 PMC5176116

[B20] Chassaing O. Desse-Berset N. Hänni C. Hughes S. Berrebi P. (2018). Microsatellite diversity of a critically endangered sturgeon, *Acipenser sturio* L. 1758, assessed from museum and archaeological tissue remains. *J. Biogeogr.* 45 1043–1053. 10.1111/jbi.13187 21483472

[B21] Cortazar-Chinarro M. Lattenkamp E. Z. Meyer-Lucht Y. Luquet E. Laurila A. Hoglund J. (2017). Drift, selection, or migration? Processes affecting genetic differentiation and variation along a latitudinal gradient in an amphibian. *BMC Evol. Biol.* 17:189 10.1186/s12862-017-1022-z PMC5557520 28806900

[B22] Craig J. F. (1996). *Pike – Biology and Exploitation.* London: Chapman & Hall.

[B23] David P. (1998). Heterozygosity–fitness correlations: new perspectives on old problems. *Heredity* 80 531–537. 10.1046/j.1365-2540.1998.00393.x 9650277

[B24] de Villemereuil P. Frichot É Bazin É François O. Gaggiotti O. E. (2014). Genome scan methods against more complex models: when and how much should we trust them? *Mol. Ecol.* 23 2006–2019. 10.1111/mec.12705 24611968

[B25] de Villemereuil P. Gaggiotti O. E. (2015). A new FST-based method to uncover local adaptation using environmental variables. *Methods Ecol. Evol.* 6 1248–1258. 10.1111/2041-210x.12418

[B26] Donadi S. Austin Å. N. Bergström U. Eriksson B. K. Hansen J. P. Jacobson P. (2017). A cross-scale trophic cascade from large predatory fish to algae in coastal ecosystems. *Proc. R. Soc. B Biol. Sci.* 284:20170045. 10.1098/rspb.2017.0045 28724727 PMC5543209

[B27] Earl D. A. vonHoldt B. M. (2012). STRUCTURE HARVESTER: a website and program for visualizing STRUCTURE output and implementing the Evanno method. *Conserv. Genet. Resour.* 4 359–361. 10.1007/s12686-011-9548-7

[B28] Edwards S. Bensch S. (2009). Looking forwards or looking backwards in avian phylogeography? A comment on Zink and Barrowclough 2008. *Mol. Ecol.* 18 2930–2933. 10.1111/j.1365-294X.2009.04270.x 19552688

[B29] Engstedt O. Engkvist R. Larsson P. (2014). Elemental fingerprinting in otoliths reveals natal homing of anadromous Baltic Sea pike (*Esox lucius* L.). *Ecol. Freshw. Fish* 23 313–321. 10.1111/eff.12082

[B30] Engstedt O. Stenroth P. Larsson P. Ljunggren L. Elfman M. (2010). Assessment of natal origin of pike (*Esox lucius*) in the Baltic Sea using Sr:Ca in otoliths. *Environ. Biol. Fishes* 89 547–555. 10.1007/s10641-010-9686-x

[B31] Eschbach E. Nolte A. W. Kohlmann K. Alos J. Schöning S. Arlinghaus R. (2019). Intraspecific population admixture of a top piscivore correlates with anthropogenic alteration of freshwater ecosystems. *bioRxiv* [Preprint]. 10.1101/677856

[B32] Evanno G. Regnaut S. Goudet J. (2005). Detecting the number of clusters of individuals using the software STRUCTURE: a simulation study. *Mol. Ecol.* 14 2611–2620. 10.1111/j.1365-294X.2005.02553.x 15969739

[B33] Ewels P. Magnusson M. Lundin S. Käller M. (2016). MultiQC: summarize analysis results for multiple tools and samples in a single report. *Bioinformatics* 32 3047–3048. 10.1093/bioinformatics/btw354 27312411 PMC5039924

[B34] Excoffier L. Lischer H. E. (2010). Arlequin suite ver 3.5: a new series of programs to perform population genetics analyses under Linux and Windows. *Mol. Ecol. Resour.* 10 564–567. 10.1111/j.1755-0998.2010.02847.x 21565059

[B35] Field D. Wills C. (1996). Long, polymorphic microsatellites in simple organisms. *Proc. Biol. Sci.* 263 209–215. 10.1098/rspb.1996.0033 8728984

[B36] Foll M. Gaggiotti O. (2008). A genome-scan method to identify selected loci appropriate for both dominant and codominant markers: a Bayesian perspective. *Genetics* 180 977–993. 10.1534/genetics.108.092221 18780740 PMC2567396

[B37] Forester B. R. Jones M. R. Joost S. Landguth E. L. Lasky J. R. (2016). Detecting spatial genetic signatures of local adaptation in heterogeneous landscapes. *Mol. Ecol.* 25 104–120. 10.1111/mec.13476 26576498

[B38] Forsman A. (2014). Effects of genotypic and phenotypic variation on establishment are important for conservation, invasion, and infection biology. *Proc. Natl. Acad. Sci. U.S.A.* 111 302–307. 10.1073/pnas.1317745111 24367109 PMC3890895

[B39] Forsman A. Tibblin P. Berggren H. Nordahl O. Koch-Schmidt P. Larson P. (2015). Pike Esox lucius as an emerging model organism for studies in ecology and evolutionary biology: a review. *J. Fish Biol.* 87 472–479. 10.1111/jfb.12712 26077107 PMC4744780

[B40] Frichot E. François O. (2015). LEA: an R package for landscape and ecological association studies. *Methods Ecol. Evol.* 6 925–929. 10.1111/2041-210x.12382

[B41] Funk W. C. Mckay J. K. Hohenlohe P. A. Allendorf F. W. (2012). Harnessing genomics for delineating conservation units. *Trends Ecol. Evol.* 27 489–496. 10.1016/j.tree.2012.05.012 22727017 PMC4185076

[B42] Goudet J. (1995). FSTAT (Version 1.2): a computer program to calculate F-statistics. *J. Hered.* 86 485–486. 10.1093/oxfordjournals.jhered.a111627

[B43] Graham C. F. Glenn T. C. McArthur A. G. Boreham D. R. Kieran T. Lance S. (2015). Impacts of degraded DNA on restriction enzyme associated DNA sequencing (RADSeq). *Mol. Ecol. Resour.* 15 1304–1315. 10.1111/1755-0998.12404 25783180

[B44] Guzinski J. Ballenghien M. Daguin-Thiébaut C. Lévêque L. Viard F. (2018). Population genomics of the introduced and cultivated Pacific kelp *Undaria pinnatifida*: marinas-not farms-drive regional connectivity and establishment in natural rocky reefs. *Evol. Appl.* 11 1582–1597. 10.1111/eva.12647 30344629 PMC6183462

[B45] Hale M. L. Burg T. M. Steeves T. E. (2012). Sampling for microsatellite-based population genetic studies: 25 to 30 Individuals per population is enough to accurately estimate allele frequencies. *PLoS One* 7:e45170. 10.1371/journal.pone.0045170 22984627 PMC3440332

[B46] Hansen M. M. Taggart J. B. Meldrup D. (1999). Development of new VNTR markers for pike and assessment of variability at di- and tetranucleotide repeat microsatellite loci. *J. Fish Biol.* 55 183–188. 10.1111/j.1095-8649.1999.tb00667.x

[B47] Hodel R. G. J. Chen S. Payton A. C. McDaniel S. F. Soltis P. Soltis D. E. (2017). Adding loci improves phylogeographic resolution in red mangroves despite increased missing data: comparing microsatellites and RAD-Seq and investigating loci filtering. *Sci. Rep.* 7:17598. 10.1038/s41598-017-16810-7 29242627 PMC5730610

[B48] Hohenlohe P. A. Bassham S. Etter P. D. Stiffler N. Johnson E. A. Cresko W. A. (2010). Population genomics of parallel adaptation in threespine stickleback using sequenced RAD tags. *PLoS Genet.* 6:e1000862. 10.1371/journal.pgen.1000862 20195501 PMC2829049

[B49] Hughes A. R. Inouye B. D. Johnson M. T. J. Underwood N. Vellend M. (2008). Ecological consequences of genetic diversity. *Ecol. Lett.* 11 609–623. 10.1111/j.1461-0248.2008.01179.x 18400018

[B50] Hutchinson W. F. (2008). The dangers of ignoring stock complexity in fishery management: the case of the North Sea cod. *Biol. Lett.* 4 693–695. 10.1098/rsbl.2008.0443 18782730 PMC2614176

[B51] Jeffries D. L. Copp G. H. Lawson Handley L. Olsén K. H. Sayer C. D. Hänfling B. (2016). Comparing RADseq and microsatellites to infer complex phylogeographic patterns, an empirical perspective in the Crucian carp, *Carassius carassius*, L. *Mol. Ecol.* 25 2997–3018. 10.1111/mec.13613 26971882

[B52] Jombart T. Devillard S. Balloux F. (2010). Discriminant analysis of principal components: a new method for the analysis of genetically structured populations. *BMC Genet.* 11:94. 10.1186/1471-2156-11-94 20950446 PMC2973851

[B53] Kopelman N. M. Mayzel J. Jakobsson M. Rosenberg N. A. Mayrose I. (2015). Clumpak: a program for identifying clustering modes and packaging population structure inferences across K. *Mol. Ecol. Resour.* 15 1179–1191. 10.1111/1755-0998.12387 25684545 PMC4534335

[B54] Larsson P. Tibblin P. Koch-Schmidt P. Engstedt O. Nilsson J. Nordahl O. (2015). Ecology, evolution, and management strategies of northern pike populations in the Baltic Sea. *Ambio* 44 451–461. 10.1007/s13280-015-0664-6 26022327 PMC4447694

[B55] Lehtonen H. Leskinen E. Selen R. Reinikainen M. (2009). Potential reasons for the changes in the abundance of pike, *Esox lucius*, in the western Gulf of Finland, 1939–2007. *Fish. Manag. Ecol.* 16 484–491. 10.1111/j.1365-2400.2009.00701.x

[B56] Lemopoulos A. Prokkola J. M. Uusi-Heikkilä S. Vasemägi A. Huusko A. Hyvärinen P. (2019). Comparing RADseq and microsatellites for estimating genetic diversity and relatedness - Implications for brown trout conservation. *Ecol. Evol.* 9 2106–2120. 10.1002/ece3.4905 30847096 PMC6392366

[B57] Ljunggren L. Sandström A. Bergström U. Mattila J. Lappalainen A. Johansson G. (2010). Recruitment failure of coastal predatory fish in the Baltic Sea coincident with an offshore ecosystem regime shift. *ICES J. Mar. Sci.* 67 1587–1595. 10.1093/icesjms/fsq109

[B58] Loiselle B. A. Sork V. L. Nason J. Graham C. (1995). Spatial genetic structure of a tropical understory shrub, *Psychotria officinalis* (Rubiaceae). *Am. J. Bot.* 82 1420–1425. 10.2307/2445869 25002460

[B59] Miller L. M. Kapuscinski A. R. (1996). Notes: microsatellite DNA markers reveal new levels of genetic variation in Northern Pike. *Trans. Am. Fish. Soc.* 125 971–977. 10.1577/1548-8659(1996)125<0971:nmdmrn>2.3.co;2

[B60] Miller L. M. Kapuscinski A. R. (1997). Historical analysis of genetic variation reveals low effective population size in a Northern Pike (Esox lucius) population. *Genetics* 147 1249–1258. 9383067 10.1093/genetics/147.3.1249 PMC1208248

[B61] Morgan T. D. Graham C. F. McArthur A. G. Raphenya A. R. Boreham D. R. Manzon R. G. (2017). Genetic population structure of the round whitefish (*Prosopium cylindraceum*) in North America: multiple markers reveal glacial refugia and regional subdivision. *Can. J. Fish. Aquat. Sci.* 75 836–849. 10.1139/cjfas-2016-0528

[B62] Muller K. (1986). Seasonal anadromous migration of the pike (*Esox lucius* L) in coastal areas of the northern Bothnian Sea. *Arch. Hydrobiol.* 107 315–330.

[B63] Nadeau N. J. Ruiz M. Salazar P. Counterman B. Medina J. A. Ortiz-Zuazaga H. (2014). Population genomics of parallel hybrid zones in the mimetic butterflies, *H. melpomene* and *H. erato*. *Genome Res.* 24 1316–1333. 10.1101/gr.169292.113 24823669 PMC4120085

[B64] Nazareno A. G. Bemmels J. B. Dick C. W. Lohmann L. G. (2017). Minimum sample sizes for population genomics: an empirical study from an Amazonian plant species. *Mol. Ecol. Resour.* 17 1136–1147. 10.1111/1755-0998.12654 28078808

[B65] Nilsson J. Flink H. Tibblin P. (2019). Predator–prey role reversal may impair the recovery of declining pike populations. *J. Anim. Ecol.* 88 927–939. 10.1111/1365-2656.12981 30895606

[B66] Nordahl O. Koch-Schmidt P. Sunde J. Yildirim Y. Tibblin P. Forsman A. (2019). Genetic differentiation between and within ecotypes of pike (*Esox lucius*) in the Baltic Sea. *Aquat. Conserv. Mar. Freshw. Ecosyst.* 29 1923–1935. 10.1002/aqc.3196

[B67] Olsson J. (2019). Past and current trends of coastal predatory fish in the Baltic Sea with a focus on perch, pike, and pikeperch. *Fishes* 4:7 10.3390/fishes4010007

[B68] Paetkau D. Slade R. Burden M. Estoup A. (2004). Genetic assignment methods for the direct, real-time estimation of migration rate: a simulation-based exploration of accuracy and power. *Mol. Ecol.* 13 55–65. 10.1046/j.1365-294X.2004.02008.x 14653788

[B69] Paris J. R. Stevens J. R. Catchen J. M. (2017). Lost in parameter space: a road map for stacks. *Methods Ecol. Evol.* 8 1360–1373. 10.1111/2041-210X.12775

[B70] Peakall R. O. D. Smouse P. E. (2006). GENALEX 6: genetic analysis in Excel. Population genetic software for teaching and research. *Mol. Ecol. Notes* 6 288–295. 10.1111/j.1471-8286.2005.01155.x 22820204 PMC3463245

[B71] Peterson B. K. Weber J. N. Kay E. H. Fisher H. S. Hoekstra H. E. (2012). Double digest RADseq: an inexpensive method for de novo SNP discovery and genotyping in model and non-model species. *PLoS One* 7:e37135. 10.1371/journal.pone.0037135 22675423 PMC3365034

[B72] Pierce R. B. Tomcko C. M. Schupp D. H. (1995). Exploitation of northern pike in seven small north-central Minnesota lakes. *N. Am. J. Fish. Manag.* 15 601–609. 10.1577/1548-8675(1995)015<0601:eonpis>2.3.co;2

[B73] Piry S. Alapetite A. Cornuet J.-M. Paetkau D. Baudouin L. Estoup A. (2004). GENECLASS2: a software for genetic assignment and first-generation migrant detection. *J. Hered.* 95 536–539. 10.1093/jhered/esh074 15475402

[B74] Plummer M. Best N. Cowles K. Vines K. (2006). CODA: convergence diagnosis and output analysis for MCMC. *R News* 6 7–11.

[B75] Pritchard J. K. Stephens M. Donnelly P. (2000). Inference of population structure using multilocus genotype data. *Genetics* 155 945–959. 10835412 10.1093/genetics/155.2.945 PMC1461096

[B76] Pujolar J. M. Jacobsen M. W. Bekkevold D. Lobón-Cervià J. Jónsson B. Bernatchez L. (2015). Signatures of natural selection between life cycle stages separated by metamorphosis in European eel. *BMC Genomics* 16:600. 10.1186/s12864-015-1754-3 26268725 PMC4535825

[B77] R Core Team (2012). *R: A Language and Environment for Statistical Computing.* Vienna: R Foundation for Statistical Computing.

[B78] Raat A. J. P. (1988). *Synopsis of Biological Data on the Northern Pike Esox lucius.* Rome: FAO.

[B79] Raj A. Stephens M. Pritchard J. K. (2014). fastSTRUCTURE: variational inference of population structure in large SNP data sets. *Genetics* 197 573–589. 10.1534/genetics.114.164350 24700103 PMC4063916

[B80] Rambaut A. Drummond A. J. Xie D. Baele G. Suchard M. A. (2018). Posterior summarization in Bayesian phylogenetics using Tracer 1.7. *Syst. Biol.* 67 901–904. 10.1093/sysbio/syy032 29718447 PMC6101584

[B81] Rannala B. Mountain J. L. (1997). Detecting immigration by using multilocus genotypes. *Proc. Natl. Acad. Sci. U.S.A.* 94 9197–9201. 10.1073/pnas.94.17.9197 9256459 PMC23111

[B82] Ravinet M. Westram A. Johannesson K. Butlin R. André C. Panova M. (2016). Shared and nonshared genomic divergence in parallel ecotypes of Littorina saxatilis at a local scale. *Mol. Ecol.* 25 287–305. 10.1111/mec.13332 26222268

[B83] Regnaut S. Lucas F. S. Fumagalli L. (2006). DNA degradation in avian faecal samples and feasibility of non-invasive genetic studies of threatened capercaillie populations. *Conserv. Genet.* 7 449–453. 10.1007/s10592-005-9023-7

[B84] Rochette N. C. Catchen J. M. (2017). Deriving genotypes from RAD-seq short-read data using Stacks. *Nat. Protoc.* 12 2640–2659. 10.1038/nprot.2017.123 29189774

[B85] Rondeau E. B. Minkley D. R. Leong J. S. Messmer A. M. Jantzen J. R. von Schalburg K. R. (2014). The genome and linkage map of the Northern Pike (*Esox lucius*): conserved synteny revealed between the salmonid sister group and the Neoteleostei. *PLoS One* 9:e102089. 10.1371/journal.pone.0102089 25069045 PMC4113312

[B86] RStudio Team (2015). *RStudio: Integrated Development for R.* Boston, MA: RStudio, Inc.

[B87] Stephenson R. L. (1999). Stock complexity in fisheries management: a perspective of emerging issues related to population sub-units. *Fish. Res.* 43 247–249. 10.1016/S0165-7836(99)00076-4

[B88] Sun J. X. Mullikin J. C. Patterson N. Reich D. E. (2009). Microsatellites are molecular clocks that support accurate inferences about history. *Mol. Biol. Evol.* 26 1017–1027. 10.1093/molbev/msp025 19221007 PMC2734136

[B89] Sunde J. Larsson P. Forsman A. (2019). Adaptations of early development to local spawning temperature in anadromous populations of pike (*Esox lucius*). *BMC Evol. Biol.* 19:148. 10.1186/s12862-019-1475-3 31331267 PMC6647320

[B90] Sunde J. Tamario C. Tibblin P. Larsson P. Forsman A. (2018a). Variation in salinity tolerance between and within anadromous subpopulations of pike (*Esox lucius*). *Sci. Rep.* 8:22 10.1038/s41598-017-18413-8 PMC5758576 29311634

[B91] Sunde J. Tibblin P. Larsson P. Forsman A. (2018b). Sex-specific effects of outbreeding on offspring quality in pike (*Esox lucius*). *Ecol. Evol.* 8 10448–10459. 10.1002/ece3.4510 30464817 PMC6238122

[B92] Sunde J. Yildirim Y. Forsman A. (2020a). *Data From: Comparing the Performance of Microsatellites and RADseq in Population Genetic Studies: Analysis of Data for Pike (*Esox lucius*) and a Synthesis of Previous Studies. NCBI Sequence Read Archive. BioProject Accession Number: PRJNA586770.* Available at: https://www.ncbi.nlm.nih.gov/Traces/study/?acc=PRJNA586770 (accessed February 13, 2020). 10.3389/fgene.2020.00218 PMC7082332 32231687

[B93] Sunde J. Yildirim Y. Tibblin P. Forsman A. (2020b). *Data From: Comparing the Performance of Microsatellites and RADseq in Population Genetic Studies: Analysis of Data for Pike (*Esox lucius*) and a Synthesis of Previous Studies, Dryad Digital Repository.* Available at: 10.5061/dryad.31zcrjdgv (accessed February 13, 2020). PMC7082332 32231687

[B94] Tibblin P. Berggren H. Nordahl O. Larsson P. Forsman A. (2016a). Causes and consequences of intra-specific variation in vertebral number. *Sci. Rep.* 6:26372. 10.1038/srep26372 27210072 PMC4876516

[B95] Tibblin P. Forsman A. Borger T. Larsson P. (2016b). Causes and consequences of repeatability, flexibility and individual fine-tuning of migratory timing in pike. *J. Anim. Ecol.* 85 136–145. 10.1111/1365-2656.12439 26412457

[B96] Tibblin P. Forsman A. Koch-Schmidt P. Nordahl O. Johannessen P. Nilsson J. (2015). Evolutionary divergence of adult body size and juvenile growth in sympatric subpopulations of a top predator in aquatic ecosystems. *Am. Nat.* 186 98–110. 10.1086/681597 26098342

[B97] Toonen R. J. Puritz J. B. Forsman Z. H. Whitney J. L. Fernandez-Silva I. Andrews K. R. (2013). ezRAD: a simplified method for genomic genotyping in non-model organisms. *PeerJ* 1:e203. 10.7717/peerj.203 24282669 PMC3840413

[B98] van Oosterhout C. Hutchinson W. F. Wills D. P. M. Shipley P. (2004). micro-checker: software for identifying and correcting genotyping errors in microsatellite data. *Mol. Ecol. Notes* 4 535–538. 10.1111/j.1471-8286.2004.00684.x

[B99] Vendrami D. L. J. Telesca L. Weigand H. Weiss M. Fawcett K. Lehman K. (2017). RAD sequencing resolves fine-scale population structure in a benthic invertebrate: implications for understanding phenotypic plasticity. *R. Soc. Open Sci.* 4:160548. 10.1098/rsos.160548 28386419 PMC5367306

[B100] Wang S. Meyer E. McKay J. K. Matz M. V. (2012). 2b-RAD: a simple and flexible method for genome-wide genotyping. *Nat. Methods* 9 808–810. 10.1038/nmeth.2023 22609625

[B101] Wei K. Wood A. R. Gardner J. P. A. (2013). Population genetic variation in the New Zealand greenshell mussel: locus-dependent conflicting signals of weak structure and high gene flow balanced against pronounced structure and high self-recruitment. *Mar. Biol.* 160 931–949. 10.1007/s00227-012-2145-9

[B102] Weir B. S. Cockerham C. C. (1984). Estimating F-statistics for the analysis of population structure. *Evolution* 38 1358–1370. 10.2307/2408641 28563791

[B103] Wennerström L. Olsson J. Ryman N. Laikre L. (2016). Temporally stable, weak genetic structuring in brackish water northern pike (*Esox lucius*) in the Baltic Sea indicates a contrasting divergence pattern relative to freshwater populations. *Can. J. Fish. Aquat. Sci.* 74 562–571. 10.1139/cjfas-2016-0039

[B104] Wilson G. A. Rannala B. (2003). Bayesian inference of recent migration rates using multilocus genotypes. *Genetics* 163 1177–1191. 12663554 10.1093/genetics/163.3.1177 PMC1462502

[B105] Wright D. Bishop J. M. Matthee C. A. von der Heyden S. (2015). Genetic isolation by distance reveals restricted dispersal across a range of life histories: implications for biodiversity conservation planning across highly variable marine environments. *Divers. Distrib.* 21 698–710. 10.1111/ddi.12302

[B106] Yıldırım Y. Anderson M. J. Hansson B. Patel S. Millar C. D. Rainey P. B. (2018a). Genetic structure of the grey side-gilled sea slug (*Pleurobranchaea maculata*) in coastal waters of New Zealand. *PLoS One* 13:e0202197. 10.1371/journal.pone.0202197 30114275 PMC6095540

[B107] Yıldırım Y. Tinnert J. Forsman A. (2018b). Contrasting patterns of neutral and functional genetic diversity in stable and disturbed environments. *Ecol. Evol.* 8 12073–12089. 10.1002/ece3.4667 30598801 PMC6303714

[B108] Zhang D.-X. Hewitt G. M. (2003). Nuclear DNA analyses in genetic studies of populations: practice, problems and prospects. *Mol. Ecol.* 12 563–584. 10.1046/j.1365-294X.2003.01773.x 12675814

[B109] Zink R. M. Groth J. G. Vázquez-Miranda H. Barrowclough G. F. (2013). Phylogeography of the California Gnatcatcher (*Polioptila californica*) using multilocus DNA sequences and ecological niche modeling: implications for conservation. *Auk* 130 449–458. 10.1525/auk.2013.12241

