# Supplementary material for: Comparing the Performance of Microsatellites and RADseq in Population Genetic Studies: Analysis of Data for Pike (Esox lucius) and a Synthesis of Previous Studies
Source: Front Genet. 2020 Mar 13;11:218. doi: 10.3389/fgene.2020.00218 (PMC7082332; doi:10.3389/fgene.2020.00218)
Supplement: Supplementary file 1 [file Data_Sheet_1.PDF]

Supplementary material

Parameter optimization

In order to choose appropriate values for the parameter settings to use in the Stacks denovo pipeline (Catchen, Hohenlohe, Bassham, Amores, & Cresko, 2013; Catchen, Amores, Hohenlohe, Cresko, & Postlethwait, 2011), we first did parameter optimization following the instructions in (Paris, Stevens, & Catchen, 2017) and (Rochette & Catchen, 2017). Thus, we run multiple runs of the pipeline with different values for three of the parameters *m* (minimum number of raw reads required to form a stack/putative allele), *M* (number of mismatches allowed between stacks/putative alleles to merge them into a putative), and *n* (number of mismatches allowed between stacks/putative loci during construction of the catalog). *m* (1-6) and *M* (0-8) were each tested by altering only the value of that specific parameter while keeping the other parameter settings constant (*m*: 3, *M*: 2, and *n*: 0). From each of the runs data on number of assembled loci, number of polymorphic loci, and number of SNPs were extracted and plotted with ggplot2 (Figure S1).

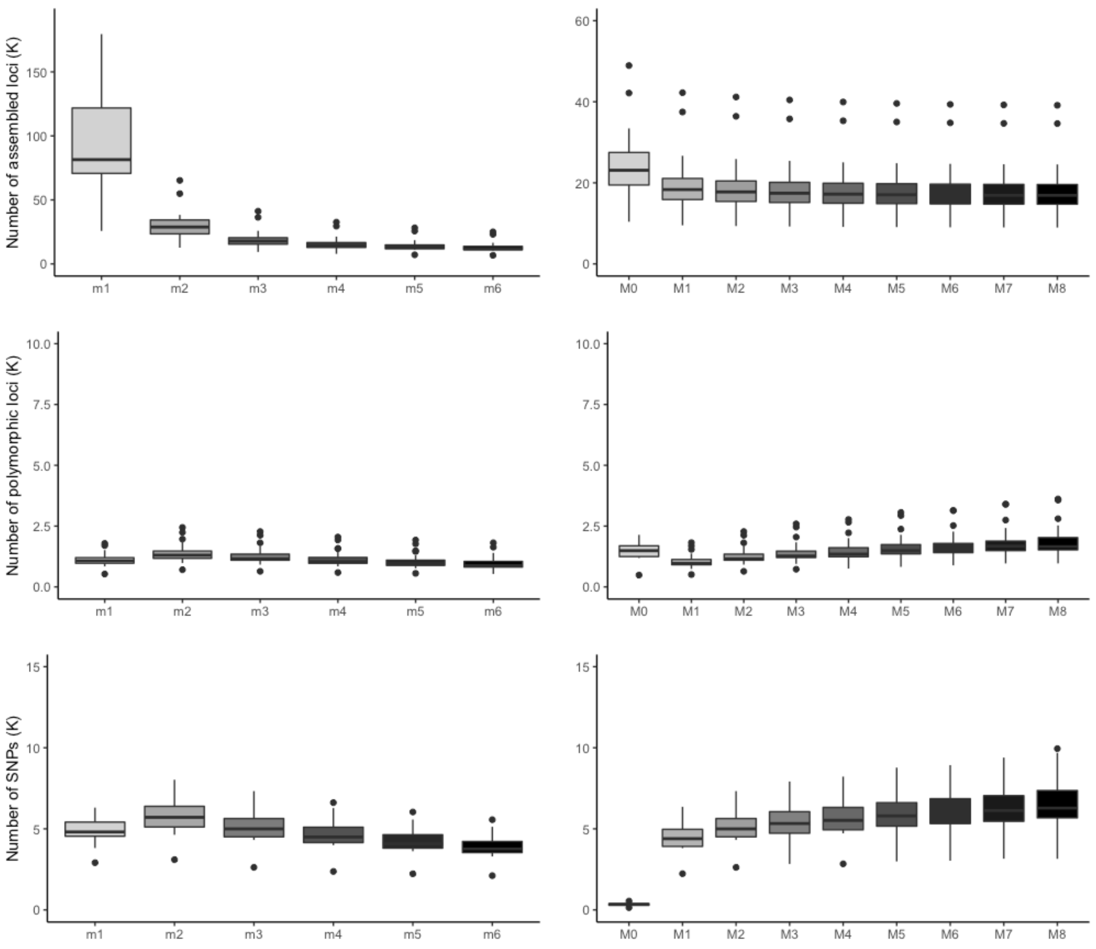

Figure S1. Results from the parameter optimization. Plots show results on number of assembled loci, number of polymorphic loci, and number of SNPs obtained from the parameter optimization of parameters *m* (minimum number of raw reads required to form a stack/putative allele) and *M* (number of mismatches allowed between stacks/putative alleles to merge them into a putative), following the instructions in (Paris et al., 2017).

For the runs testing *m* mean primary coverage and mean merged coverage for each sample were also extracted and plotted (Figure S2A). Further *n* (1-9) was tested by altering the value of that specific setting and in addition keeping *M* = *n*. For these runs data on percentage of loci containing different numbers of SNPs (0 – 10 SNPs separately, and loci with >10 SNPs grouped) were extracted and plotted (Figure S2B). The plots were inspected to determine which settings to use for the integrated approach (Paris et al., 2017), and the parameters we chose to use in the integrated approach were *m* = 3, *M* = 3, and *n* = 3.

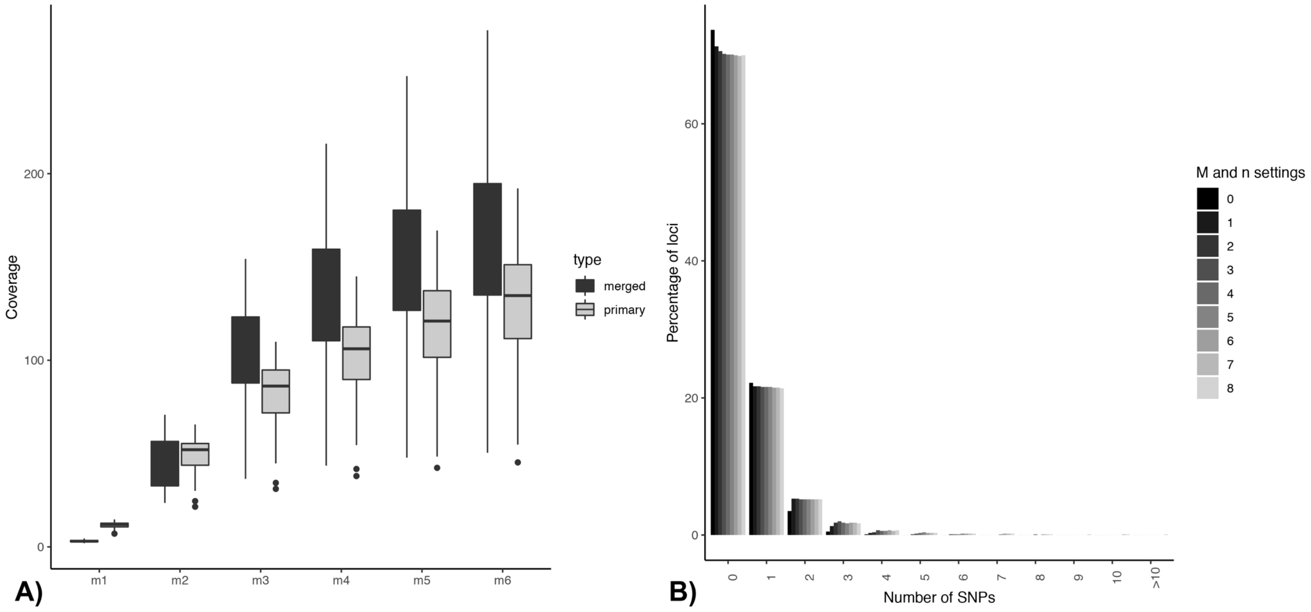

Figure S2. Results obtained from the parameter optimization. A) Data on primary and merged coverage for different setting of parameter m (minimum number of raw reads required to form a stack/putative allele) following the instructions in (Paris et al., 2017). B) Data on percentage of loci with different number of SNPs for different settings of M (number of mismatches allowed between stacks/putative alleles to merge them into a putative) and n (number of mismatches allowed between stacks/putative loci during construction of the catalog) following the instuctions in (Rochette & Catchen, 2017).

To evaluate if the chosen parameters seemed to be appropriate to use, we ran two extra runs of the Stacks denovo pipeline, one with the settings chosen based on the parameter optimization and one with the default settings (*m* = 3, *M* = 2, *n* = 0), extracted data on number of assembled loci, number of polymorphic loci and number of SNPs, and plotted the results (Figure S3). The plots indicated that the performance of the pipeline with the parameter values chosen based on the parameter optimization was somewhat better than that of the default settings (slightly higher number of polymorphic loci and number of SNPs), and we therefore chose to proceed with those parameter settings.

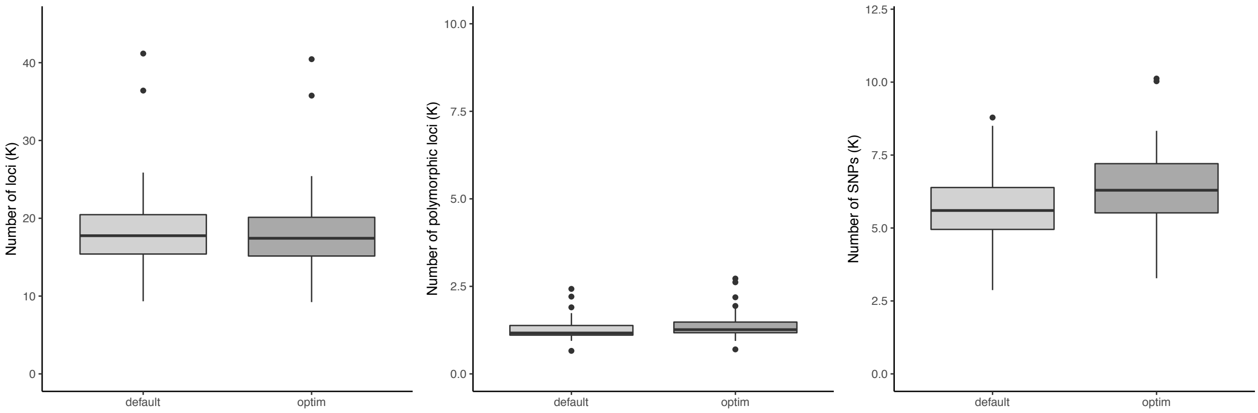

Figure S3. Comparison of default and optimized parameter settings in the Stacks pipeline. Number of assembled loci, number of polymorphic loci and number of SNPs obtained from two runs of the Stacks denovo pipeline. The left box in each plot (in light grey) shows the results from the run with the default settings ($m = 3$, $M = 2$, $n = 0$), and the right box in each plot shows the results from the run with the parameter settings chosen based on the parameter optimization ($m = 3$, $M = 3$, $n = 3$).

Supplementary figures

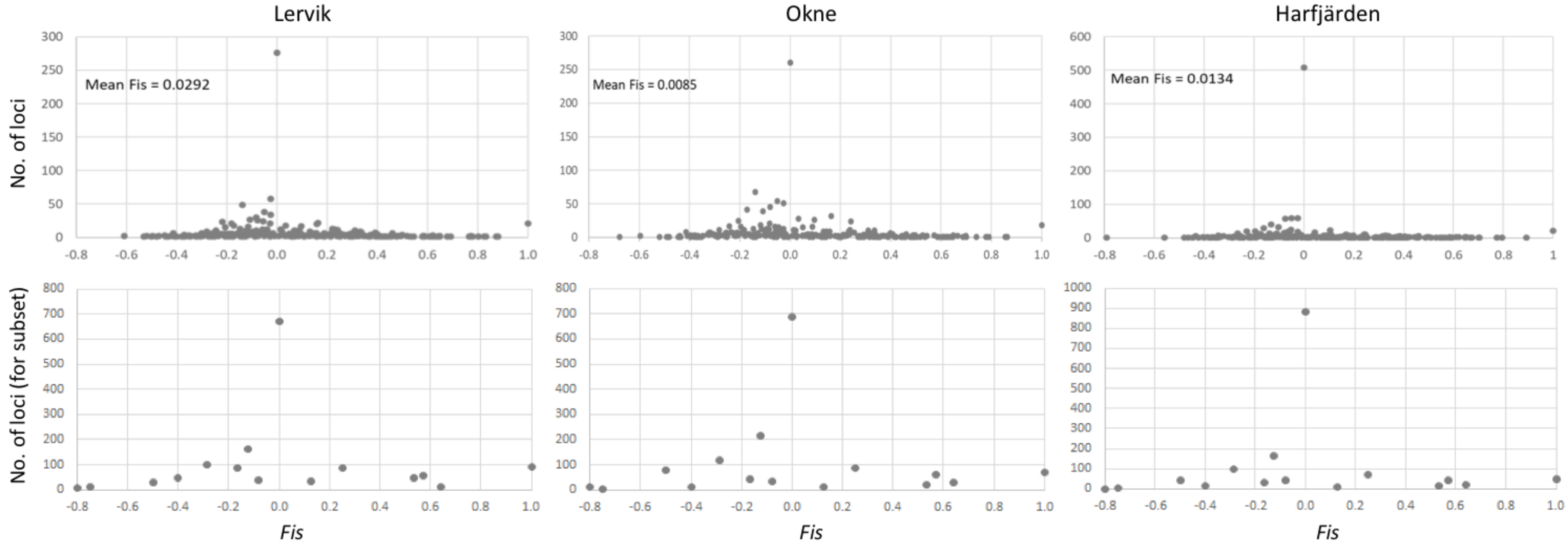

Figure S4. Distribution of *Fis* for the three populations. Top row show *Fis* distribution for the entire dataset ($N = 64$) and bottom row show the *Fis* distribution for a subset of 10 individuals from each population ($N = 30$).

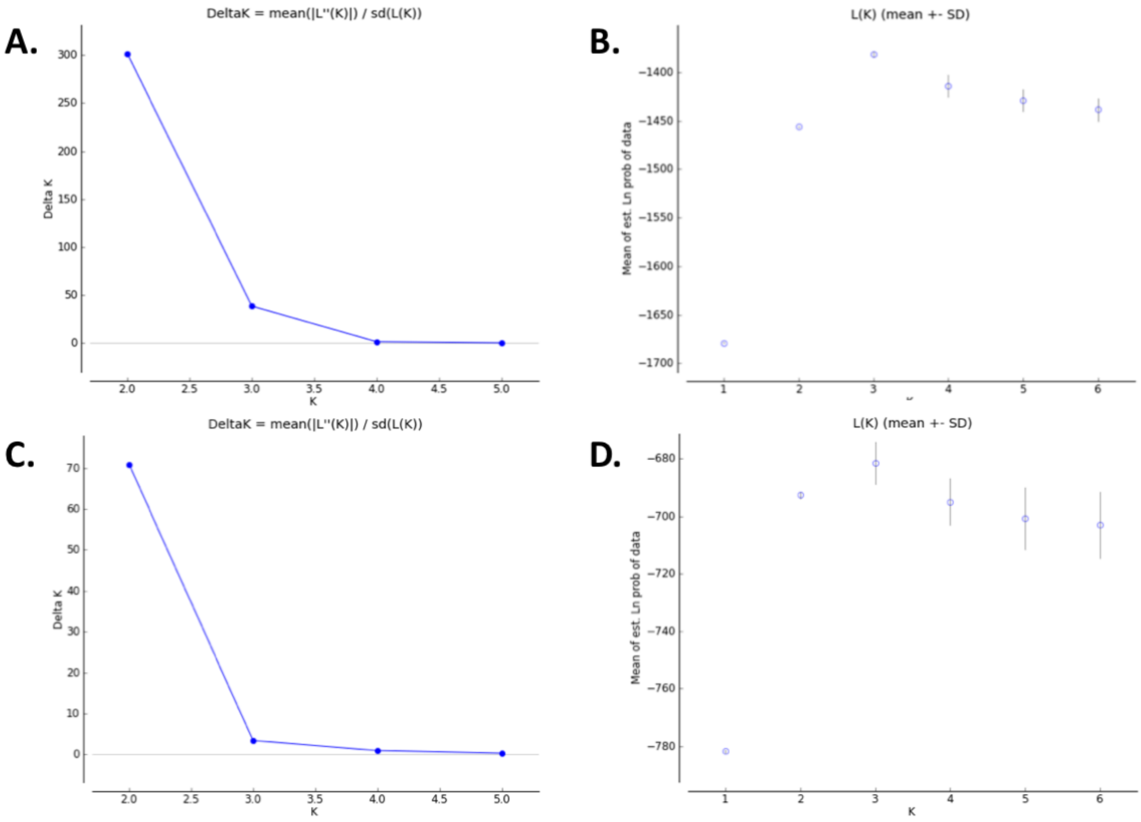

Figure S5. Results of Bayesian clustering analysis for microsatellite data with sampling location introduced as *a priori*. Upper panel show the results for the full microsatellite dataset ($N = 64$) and the lower panel the results for the subset microsatellite datasets ($N = 30$). Left plots (A and C) show ΔK versus K, and right plots (B and D) the mean posterior probability of the data L(K) (±SD).

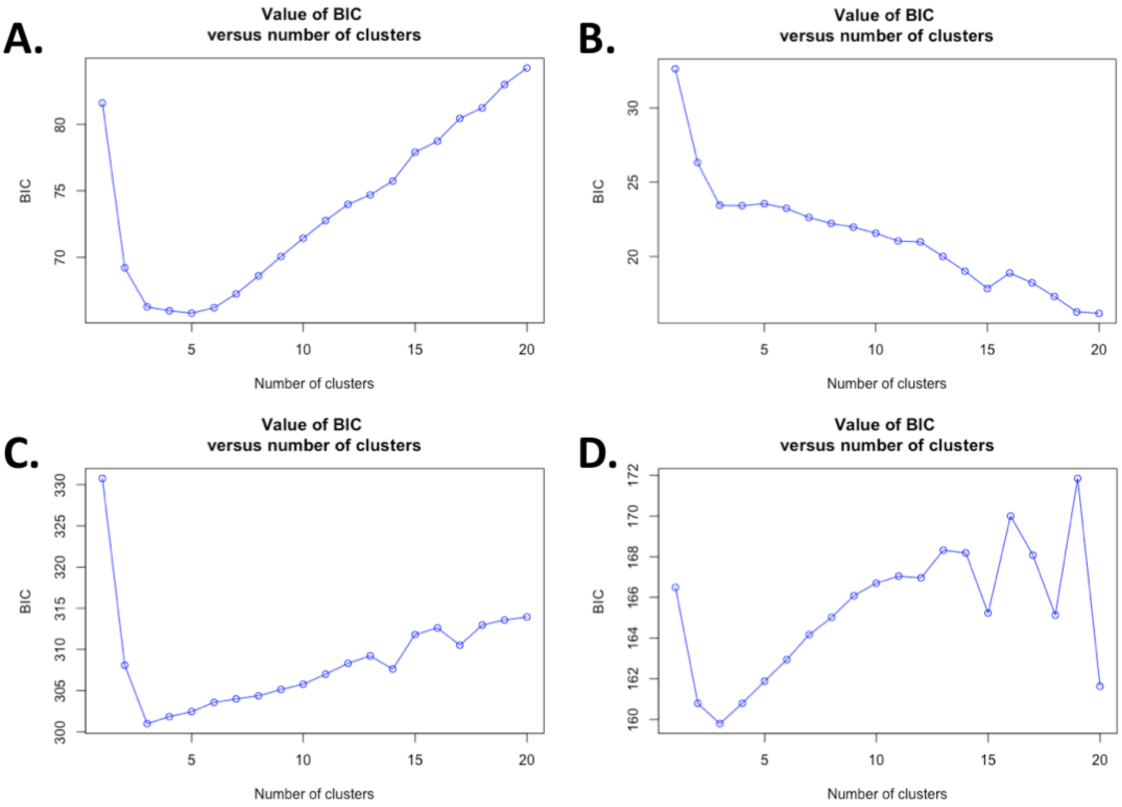

Figure S6. Bayesian Information Criterion (BIC) for different numbers of clusters in Discriminant Analysis of Principal Components (DAPC). Plots are based on A) full microsatellite dataset (10 loci, $N = 64$), B) subset microsatellite dataset (10 loci, $N = 30$), C) full RADseq SNP dataset (1580 biallelic SNPs, $N = 64$), and D) subset RADseq SNP dataset (1670 biallelic SNPs, $N = 30$).

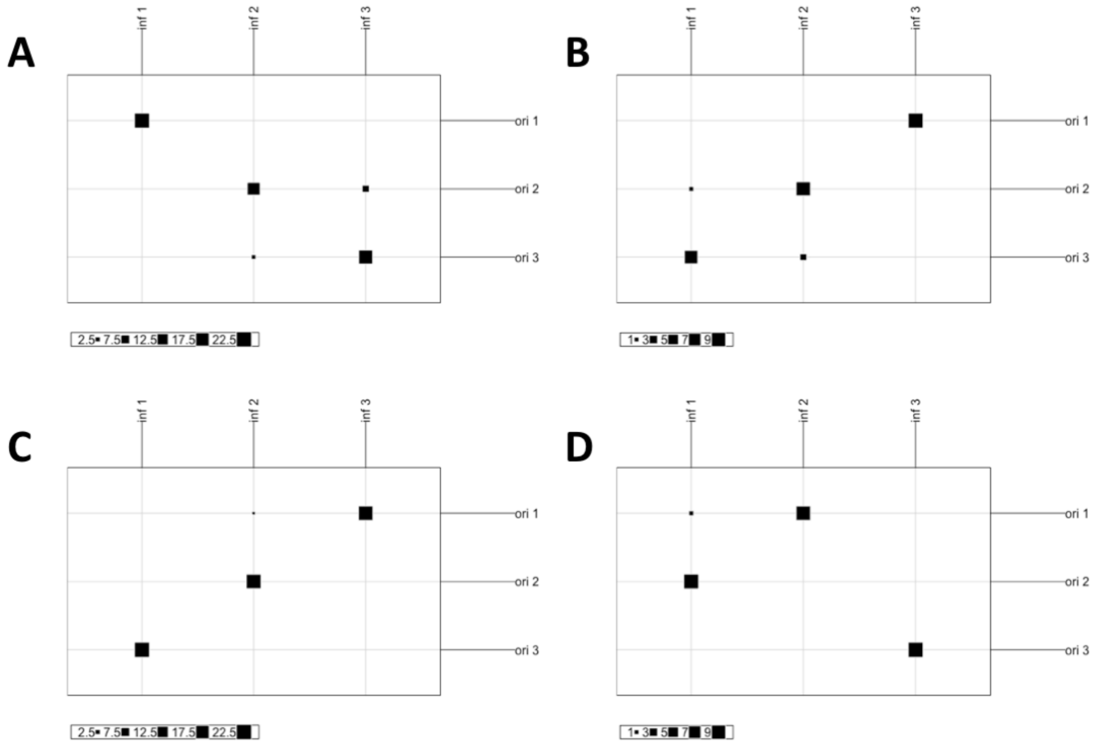

Figure S7. Miss-classified individuals in DAPC. "ori" indicate origin of individuals, and "inf" indicate the inferred cluster. Plots are based on A) full microsatellite dataset (10 loci, $N = 64$, mis-classified individuals = 7), B) subset microsatellite dataset (10 loci, $N = 30$, mis-classified individuals = 3), C) full RADseq SNP dataset (1580 biallelic SNPs, $N = 64$, mis-classified individuals = 1), and D) subset RADseq SNP dataset (1670 biallelic SNPs, $N = 30$, mis-classified individuals = 1).

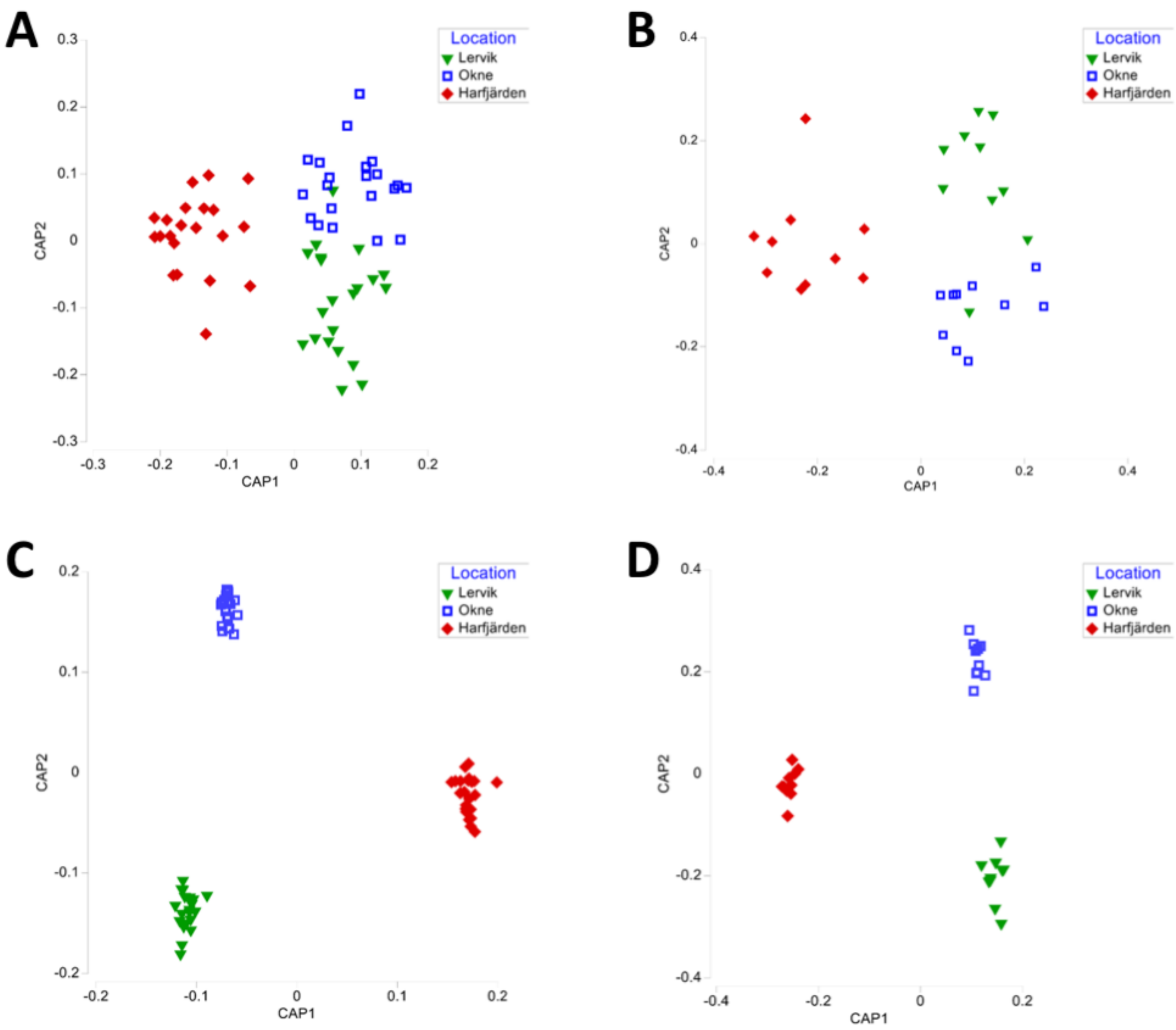

Figure S8. Visualization of Canonical Analysis of Principal (CAP) coordinates for both full (*N* = 64) and subset (*N* = 30) datasets for both microsatellites and RADseq SNPs. Plots show A) full microsatellite dataset (10 loci, *N* = 64), B) subset microsatellite dataset (10 loci, *N* = 30), C) full RADseq SNP dataset (1580 biallelic SNPs, *N* = 64), and D) subset RADseq SNP dataset (1670 biallelic SNPs, *N* = 30).

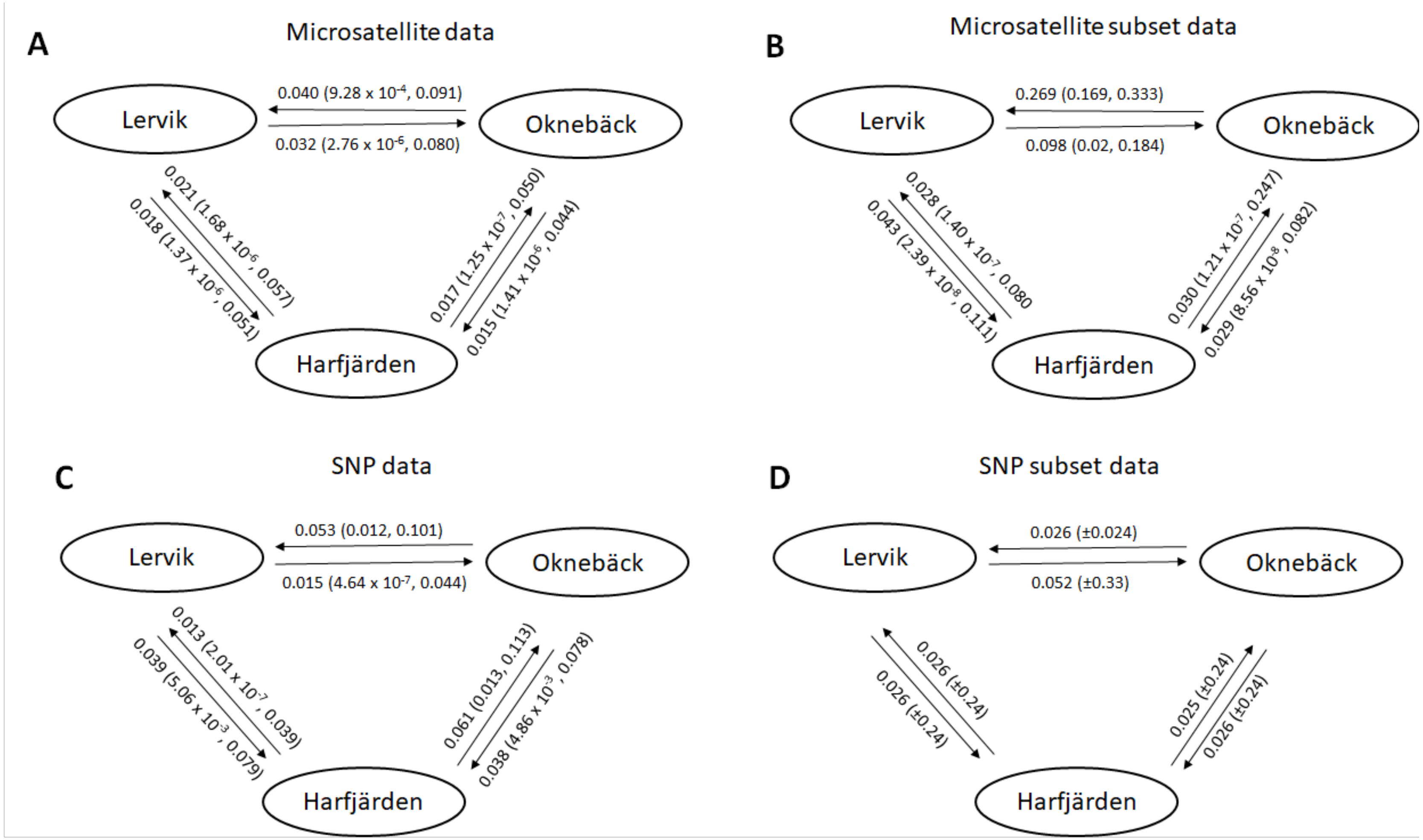

Figure S9. Estimated recent gene flow between the three populations based on microsatellites and RADseq SNP data. The results of BAYESASS analysis showing the proportion of the populations derived from the other populations. (95% confidence intervals). Plots show results for A) full microsatellite dataset (*N* = 64), B) subset microsatellite dataset (*N* = 10), C) full RADseq SNP dataset (*N* = 64), and D) subset RAdseq SNP dataset (*N* = 10). Arrows show the direction of the gene flow.

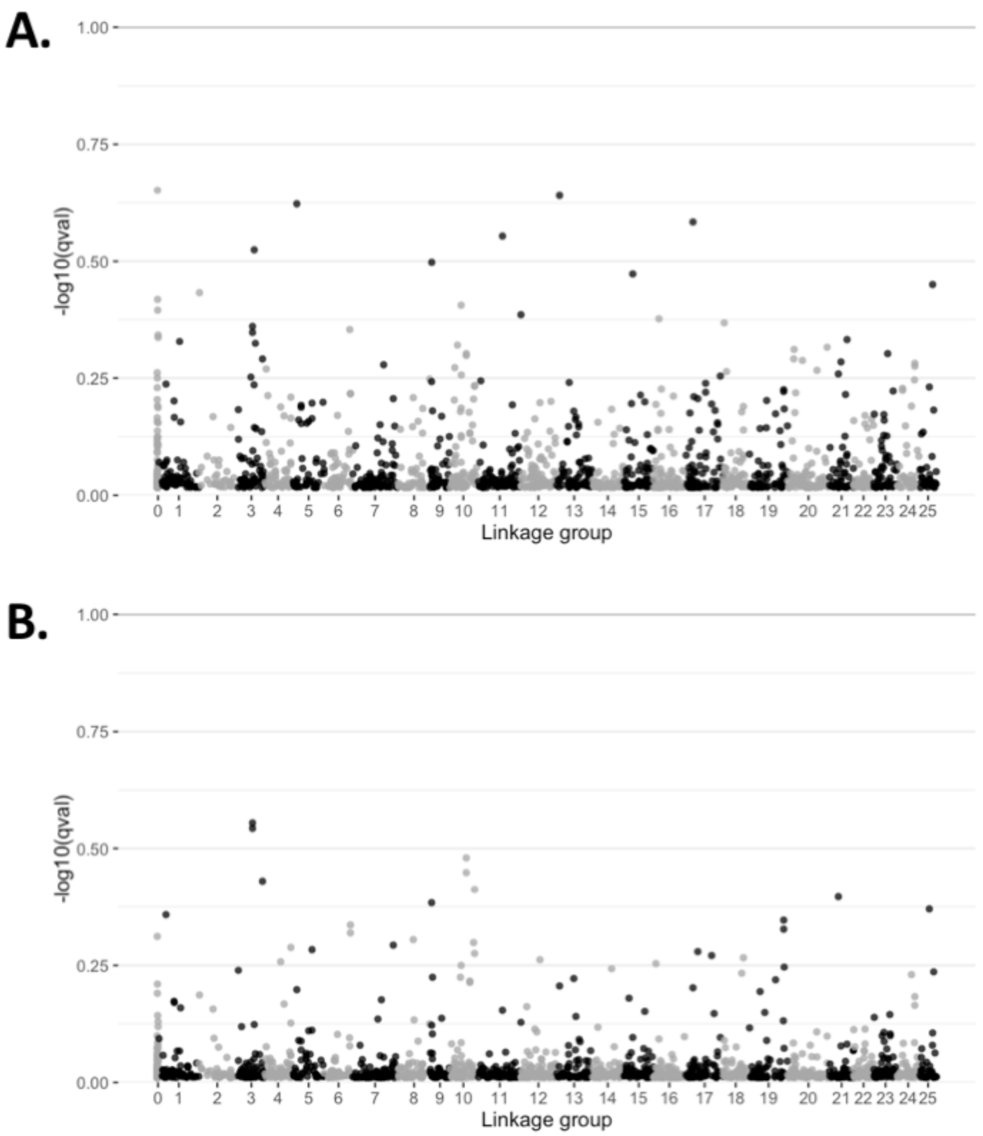

Figure S10. Identification of outlier SNPs. Plots showing the results from a Bayesian test of SNPs correlated with environmental variables (BayeScEnv). Plots show the -log10 *q*-values for each SNP distributed along the linkage groups for two environmental variables A) salinity in the spawning ground, and B) temperature at initiation of spawning.

Supplementary tables

Table S1. Estimation of the null allele frequencies for the full microsatellite dataset (10 loci, $N = 64$) using the ENA algorithm described in Chapuis and Estoup (2006).

**Locus**	**Population**	**Null allele frequency**	**Locus**	**Population**	**Null allele frequency**
Elu87	Lervik	0.08658	Elu64	Lervik	0.07086
Elu87	Okne	0.06727	Elu64	Okne	0
Elu87	Harfjärden	0.04549	Elu64	Harfjärden	0.00001
Elu19	Lervik	0	Elu2	Lervik	0.00001
Elu19	Okne	0.03406	Elu2	Okne	0.05709
Elu19	Harfjärden	0.00583	Elu2	Harfjärden	0.00001
Elu76	Lervik	0	Elu78	Lervik	0
Elu76	Okne	0	Elu78	Okne	0.00001
Elu76	Harfjärden	0	Elu78	Harfjärden	0
Elu51	Lervik	0.09559	Elu37	Lervik	0
Elu51	Okne	0.00001	Elu37	Okne	0.09442
Elu51	Harfjärden	0.08875	Elu37	Harfjärden	0.00002
Elu6	Lervik	0.03138	Elu276	Lervik	0
Elu6	Okne	0.02656	Elu276	Okne	0.05297
Elu6	Harfjärden	0.16843	Elu276	Harfjärden	0

Table S2. Estimated global F_{ST} and pairwise F_{ST} for each microsatellite loci with and without ENA correction described in Chapuis and Estoup (2007). Data for the full microsatellite dataset (10 loci, $N = 64$).

All loci

F_{ST} not using ENA

Population	Harfjärden	Lervik
Lervik	0.272	
Okne	0.252	0.106

F_{ST} using ENA

Population	Harfjärden	Lervik
Lervik	0.270	
Okne	0.253	0.105

Elu87

F_{ST} not using ENA

Population	Harfjärden	Lervik
Lervik	-0.007	
Okne	0.042	0.054

F_{ST} using ENA

Population	Harfjärden	Lervik
Lervik	-0.000	
Okne	0.044	0.051

Elu64

F_{ST} not using ENA

Population	Harfjärden	Lervik
Lervik	0.273	
Okne	0.283	0.130

F_{ST} using ENA

Population	Harfjärden	Lervik
Lervik	0.261	
Okne	0.283	0.121

Elu19

F_{ST} not using ENA

Population	Harfjärden	Lervik
Lervik	0.201	
Okne	0.267	0.078

F_{ST} using ENA

Population	Harfjärden	Lervik
Lervik	0.199	
Okne	0.270	0.080

Elu2

F_{ST} not using ENA

Population	Harfjärden	Lervik
Lervik	0.638	
Okne	0.143	0.333

F_{ST} using ENA

Population	Harfjärden	Lervik
Lervik	0.638	
Okne	0.163	0.332

Elu76

F_{ST} not using ENA

Population	Harfjärden	Lervik
Lervik	0.227	
Okne	0.141	0.050

F_{ST} using ENA

Population	Harfjärden	Lervik
Lervik	0.227	
Okne	0.141	0.050

Elu78

F_{ST} not using ENA

Population	Harfjärden	Lervik
Lervik	-0.009	
Okne	0.000	0.022

F_{ST} using ENA

Population	Harfjärden	Lervik
Lervik	-0.009	
Okne	0.000	0.022

Elu51

F_{ST} not using ENA

Population	Harfjärden	Lervik
Lervik	0.030	
Okne	0.256	0.085

F_{ST} using ENA

Population	Harfjärden	Lervik
Lervik	0.0250	
Okne	0.266	0.111

Elu37

F_{ST} not using ENA

Population	Harfjärden	Lervik
Lervik	0.213	
Okne	0.381	0.061

F_{ST} using ENA

Population	Harfjärden	Lervik
Lervik	0.213	
Okne	0.384	0.059

Elu6

F_{ST} not using ENA

Population	Harfjärden	Lervik
Lervik	0.196	
Okne	0.185	0.083

F_{ST} using ENA

Population	Harfjärden	Lervik
Lervik	0.190	
Okne	0.179	0.081

Elu276

F_{ST} not using ENA

Population	Harfjärden	Lervik
Lervik	0.511	
Okne	0.525	0.084

F_{ST} using ENA

Population	Harfjärden	Lervik
Lervik	0.511	
Okne	0.514	0.075

Table S3. Results from Bayescan analysis of microsatellite loci to test for signs of selection, which indicated that all loci were neutral (*q*-value > 0.05). Data for the full microsatellite dataset (10 loci, $N = 64$).

Locus	*P*-value	*q*-value	alpha	F_{ST}
Elu87	0.11	0.71	-0.08	0.18
Elu19	0.04	0.89	0.01	0.19
Elu76	0.06	0.87	-0.01	0.19
Elu51	0.08	0.83	-0.02	0.19
Elu6	0.47	0.53	-0.39	0.14
Elu64	0.05	0.88	-0.01	0.19
Elu2	0.10	0.80	0.06	0.20
Elu78	0.10	0.77	-0.07	0.18
Elu37	0.06	0.87	0.02	0.19
Elu276	0.08	0.84	0.04	0.20

Locus: microsatellite locus. *q*-value: FDR-corrected *P*-value.

Table S4. Genetic diversity indices for the full microsatellite dataset (10 loci, *N* = 64) at each locus for each population

Population	Locus	*N*	*Na*	AR	PA	*Ho*	*He*	*P*-value	Fis
**Harfjärden**	**Elu87**	22	2	2.00	-	0.41	0.49	0.65	0.14
	**Elu19**	22	2	2.00	-	0.41	0.43	1.00	0.02
	**Elu76**	22	4	3.91	1	0.32	0.33	0.54	-0.003
	**Elu51**	22	2	2.00	-	0.36	0.51	0.22	0.27
	**Elu6**	22	15	14.40	11	0.55	0.90	0.00	0.38
	**Elu64**	22	4	4.00	-	0.59	0.59	0.87	-0.03
	**Elu2**	22	2	2.00	-	0.14	0.13	1.00	-0.07
	**Elu78**	22	3	2.99	1	0.41	0.35	1.00	-0.20
	**Elu37**	22	3	2.99	-	0.41	0.43	0.76	0.02
	**Elu276**	22	4	3.99	1	0.41	0.39	0.73	-0.07
	**Mean (SE)**	22	4.1 (1.24)	4.78	-	0.56 (0.18)*	0.59 (0.19)	-	0.12
**Lervik**	**Elu87**	21	4	4.00	-	0.38	0.55	0.19	0.30
	**Elu19**	21	5	4.95	1	0.76	0.70	0.65	-0.12
	**Elu76**	21	5	4.95	1	0.81	0.66	0.18	-0.26
	**Elu51**	21	2	2.00	-	0.29	0.42	0.27	0.3
	**Elu6**	20	11	11.00	5	0.65	0.66	0.68	-0.008
	**Elu64**	21	5	4.91	1	0.48	0.65	0.21	0.25
	**Elu2**	21	3	3.00	-	0.48	0.51	0.74	0.04
	**Elu78**	21	2	2.00	-	0.29	0.25	1.00	-0.17
	**Elu37**	21	6	5.91	2	0.76	0.77	0.55	-0.01
	**Elu276**	21	2	2.00	-	0.76	0.50	0.03	-0.56
	**Mean (SE)**	21	4.5 (0.60)	4.02	-	0.57 (0.21)*	0.57 (0.15)	-	0.003
**Okne**	**Elu87**	21	6	5.91	2	0.57	0.67	0.09	0.13
	**Elu19**	21	6	6.00	2	0.71	0.79	0.68	0.08
	**Elu76**	21	5	4.95	2	0.76	0.70	0.55	-0.11
	**Elu51**	21	2	2.00	-	0.19	0.18	1.00	-0.11
	**Elu6**	20	8	8.00	5	0.70	0.70	0.68	-0.02
	**Elu64**	21	4	4.00	-	0.67	0.64	0.90	-0.07
	**Elu2**	21	3	3.00	-	0.48	0.53	0.02	0.09
	**Elu78**	21	3	3.00	1	0.43	0.44	0.75	0.01
	**Elu37**	21	7	6.95	3	0.62	0.76	0.03	0.17
	**Elu276**	21	4	4.00	1	0.43	0.43	0.10	-0.01
	**Mean (SE)**	21	4.8 (0.66)	4.47	-	0.40 (0.12)*	0.45 (0.20)	-	0.05

N: number of samples. *Na*: total number of alleles. H_O: observed heterozygosity. H_E: expected heterozygosity. PA: the number of private alleles. *Fis*: fxation index. SE: standard error. * $P < 0.05$.

Table S5. Detection of first generation migrants for both full datasets (microsatellite and RADseq SNPs). The individuals identified as potential migrants ($P < 0.01$) are indicated in **red**, and the most likely population in *green*. ID: individual identifier.

ID	Population origin	Microsatellite -LOG (LH/Lmax)	Microsatellite Probability	Microsatellite Harfjärden -Log(L)	Microsatellite Lervik -Log(L)	Microsatellite Okne -Log(L)	SNP -LOG (LH/Lmax)	SNP Probability	SNP Harfjärden -Log(L)	SNP Lervik -Log(L)	SNP Okne -Log(L)
HF1	Harfjärden	0.000	0.50053	6.080	14.515	19.438	0.000	0.500	744.341	717.740	385.203
HF3	Harfjärden	0.000	0.50061	7.225	14.916	18.195	0.000	0.500	686.609	682.920	367.789
HF5	Harfjärden	0.000	0.50042	6.267	18.516	18.000	0.000	0.500	729.139	709.741	363.018
HF6	Harfjärden	0.000	0.50043	5.998	17.369	18.087	0.000	0.500	725.257	719.759	364.374
HF7	Harfjärden	0.000	0.50010	10.263	11.040	17.429	0.000	0.500	723.509	718.739	358.841
HF9	Harfjärden	0.000	0.50050	9.446	18.801	19.434	0.000	0.500	743.184	731.165	365.833
HF10	Harfjärden	0.000	0.50057	8.712	14.489	17.317	0.000	0.500	756.289	749.596	385.764
HF11	Harfjärden	0.000	0.50045	8.295	14.175	19.853	0.000	0.500	728.687	722.803	356.166
HF12	Harfjärden	0.000	0.50039	4.521	15.699	18.066	0.000	0.500	738.194	753.133	335.168
HF13	Harfjärden	0.000	0.50044	8.128	15.719	15.956	0.000	0.500	710.911	696.987	375.299
HM1	Harfjärden	0.000	0.50059	5.861	15.850	17.014	0.000	0.500	740.848	723.746	372.557
HM2	Harfjärden	0.000	0.50059	6.586	19.179	16.976	0.000	0.500	747.816	742.565	360.770
HM3	Harfjärden	0.000	0.50053	8.805	15.571	16.086	0.000	0.500	745.920	738.741	363.461
HM4	Harfjärden	0.000	0.50046	6.268	16.631	18.406	0.000	0.500	751.832	712.299	361.619
HM5	Harfjärden	0.000	0.50035	6.212	17.482	17.177	0.000	0.500	771.228	761.366	367.121
HM6	Harfjärden	0.000	0.50053	10.163	18.512	19.187	0.000	0.500	728.544	726.534	335.386
HM7	Harfjärden	0.000	0.50072	7.807	17.802	18.897	0.000	0.500	785.131	768.483	363.814
HM8	Harfjärden	0.000	0.50054	8.808	17.449	15.016	0.000	0.500	776.889	779.747	382.481
HM9	Harfjärden	0.000	0.50049	6.922	17.712	18.750	0.000	0.500	746.928	721.809	368.577
HM10	Harfjärden	0.000	0.50066	6.102	15.274	16.225	0.000	0.500	721.226	702.659	369.459
HM11	Harfjärden	0.000	0.50036	6.070	18.235	17.096	0.000	0.500	788.071	750.312	337.185
HM12	Harfjärden	0.000	0.50052	4.521	15.699	18.066	0.000	0.500	735.696	746.422	333.909

		Microsatellite					SNP				
**ID**	**Population origin**	**-LOG (LH/Lmax)**	**Probability**	**Harfjärden -Log(L)**	**Lervik -Log(L)**	**Okne -Log(L)**	**-LOG (LH/Lmax)**	**Probability**	**Harfjärden -Log(L)**	**Lervik -Log(L)**	**Oknebäck -Log(L)**
LF1	Lervik	0.000	0.51330	19.824	6.299	8.428	0.000	0.501	871.793	469.752	626.931
LF2	Lervik	0.000	0.51441	13.725	7.146	12.360	0.000	0.500	881.632	489.388	584.904
LF3	**Lervik**	**4.174**	0.00004	18.268	10.807	*6.633*	**19.358**	**0.000**	866.432	540.096	*520.738*
LF4	Lervik	0.000	0.51387	16.434	7.806	17.096	0.000	0.501	841.662	483.418	595.830
LF6	Lervik	0.000	0.51409	15.705	7.084	9.884	0.000	0.500	811.025	474.729	601.826
LF7	Lervik	0.000	0.51365	17.793	5.974	13.471	0.000	0.501	829.212	436.226	592.892
LF8	Lervik	0.000	0.51413	18.873	9.190	13.276	0.000	0.502	862.297	436.445	617.574
LF9	Lervik	0.000	0.51446	15.344	5.632	11.392	0.000	0.500	838.411	446.312	599.503
LF11	Lervik	0.000	0.51796	15.423	5.659	11.443	0.000	0.501	840.770	439.883	598.484
LM1	Lervik	0.000	0.51344	23.653	9.123	16.335	0.000	0.501	872.586	466.647	607.436
LM2	Lervik	0.279	0.02191	18.349	10.789	10.509	0.000	0.500	942.333	488.812	661.190
LM3	Lervik	0.000	0.51416	14.070	8.936	12.500	0.000	0.500	850.787	462.284	577.306
LM4	Lervik	0.000	0.51306	18.294	12.569	15.359	0.000	0.500	865.582	487.296	584.663
LM5	Lervik	0.000	0.51350	20.466	8.973	12.219	0.000	0.502	846.131	448.756	582.652
LM6	Lervik	0.000	0.51415	19.815	8.293	16.890	0.000	0.500	885.268	476.937	618.636
LM7	Lervik	0.000	0.51437	20.754	7.188	13.405	0.000	0.500	520.929	300.374	400.295
LM8	Lervik	0.000	0.51367	20.662	9.601	14.642	0.000	0.501	893.638	460.609	607.667
LM9	Lervik	0.000	0.51430	11.187	8.179	10.291	0.000	0.500	864.752	442.186	621.330
LM10	Lervik	0.000	0.51356	22.162	9.107	15.578	0.000	0.500	845.134	489.887	592.169
LM11	Lervik	0.000	0.51307	22.743	10.280	11.869	0.000	0.501	930.506	497.574	579.437
LM12	Lervik	0.000	0.51278	14.851	9.206	12.591	0.000	0.501	810.402	459.880	572.738

ID	Population origin	Microsatellite -Log (LH/Lmax)	Probability	Harfjärden -Log(L)	Lervik -Log(L)	Oknebäck -Log(L)	SNP -LOG (LH/Lmax)	Probability	Harfjärden -Log(L)	Lervik -Log(L)	Oknebäck -Log(L)
OF1	Oknebäck	0.000	0.50426	17.987	9.571	6.916	0.000	0.500	850.093	586.476	462.772
OF2	Oknebäck	0.000	0.50450	15.131	10.099	7.193	0.000	0.500	897.878	617.032	486.728
OF3	Oknebäck	0.000	0.50517	14.985	11.971	7.613	0.000	0.500	813.525	564.746	472.198
OF4	Oknebäck	0.000	0.50471	16.150	10.648	9.585	0.000	0.500	891.582	607.048	483.494
OF5	Oknebäck	0.000	0.50486	14.599	11.776	7.662	0.000	0.500	863.594	597.969	480.631
OF6	Oknebäck	0.000	0.50542	19.612	12.093	7.064	0.000	0.500	834.677	592.093	467.693
OF7	Oknebäck	0.000	0.50464	21.548	12.443	8.836	0.000	0.500	917.011	654.661	496.824
OF8	**Oknebäck**	**1.720**	**0.00050**	24.411	*14.217*	15.937	0.000	0.500	921.251	637.948	493.250
OF9	Oknebäck	0.000	0.50492	24.262	17.240	8.976	0.000	0.500	879.812	599.253	482.661
OF10	Oknebäck	0.000	0.50474	20.877	14.308	9.024	0.000	0.500	869.533	618.738	480.553
OF11	Oknebäck	0.000	0.50493	16.769	10.177	8.190	0.000	0.500	794.700	556.004	459.384
OM1	Oknebäck	0.000	0.50464	12.863	9.692	7.412	0.000	0.500	836.232	576.122	461.917
OM2	Oknebäck	0.000	0.50485	19.301	14.618	12.135	0.000	0.500	860.232	617.351	476.129
OM3	Oknebäck	0.000	0.50536	26.377	16.911	12.773	0.000	0.500	843.830	579.483	466.954
OM4	Oknebäck	0.000	0.51099	14.530	11.072	6.027	0.000	0.500	916.821	616.721	492.509
OM5	Oknebäck	0.000	0.50473	21.148	15.391	10.097	0.000	0.500	903.223	616.329	481.709
OM6	Oknebäck	0.000	0.50443	23.338	16.516	9.962	0.000	0.500	864.263	635.343	471.996
OM7	Oknebäck	0.000	0.50391	23.881	13.265	10.781	0.000	0.500	839.611	608.996	471.005
OM8	Oknebäck	0.000	0.50497	21.909	12.847	9.437	0.000	0.500	860.868	580.576	443.383
OM9	Oknebäck	0.000	0.50516	22.924	14.119	9.854	0.000	0.500	887.921	613.392	491.177
OM10	Oknebäck	0.000	0.50465	23.275	11.717	8.162	0.000	0.500	814.592	577.450	471.846

Table S6. Detection of first generation migrants for the microsatellite and RADseq SNP data for a subset of the same 10 individuals from each population. The induviduals identified as potential migrants ($P < 0.01$) are indicated in **red**, and the most likely population in *green*. ID: individual identifier.

		**Microsatellites for subset of individuals**					**SNPs for subset of individuals**				
**ID**	**Population origin**	**-Log (LH/Lmax)**	**Probability**	**Harfjärden -Log(L)**	**Lervik -Log(L)**	**Oknebäck -Log(L)**	**-Log (LH/Lmax)**	**Probability**	**Harfjärden -Log(L)**	**Lervik -Log(L)**	**Oknebäck -Log(L)**
HF3	Harfjärden	0.000	0.501	7.090	14.988	16.085	0.000	0.500	683.487	690.735	402.165
HF5	Harfjärden	0.000	0.500	6.826	18.043	17.160	0.000	0.500	720.248	710.893	388.179
HF7	Harfjärden	0.000	0.500	10.047	11.884	15.385	0.000	0.500	725.259	734.077	378.021
HF10	Harfjärden	0.000	0.501	9.778	14.634	14.648	0.000	0.500	752.434	759.216	430.39
HF11	Harfjärden	0.000	0.500	9.652	16.087	18.522	0.000	0.500	732.084	732.951	375.881
HM12	Harfjärden	0.000	0.500	5.799	15.006	16.192	0.000	0.500	741.887	744.376	388.321
HM3	Harfjärden	0.000	0.500	8.304	15.462	15.149	0.000	0.500	751.877	723.107	381.631
HM4	Harfjärden	0.000	0.500	7.900	16.180	16.849	0.000	0.500	757.896	763.646	385.727
HM5	Harfjärden	0.000	0.500	7.538	16.415	15.507	0.000	0.500	771.083	756.493	388.684
HM7	Harfjärden	0.000	0.501	7.003	17.496	17.534	0.000	0.500	732.862	754.848	382.913
LF1	Lervik	0.000	0.525	18.175	6.773	7.526	0.000	0.506	507.973	667.719	863.682
LF3	**Lervik**	**2.295**	**0.000**	17.957	9.868	*7.574*	**35.053**	**0.005**	590.73	*555.678*	870.16
LF6	Lervik	0.000	0.521	14.723	7.993	9.521	0.000	0.506	522.743	642.319	835.092
LF9	Lervik	0.000	0.523	14.365	7.841	10.542	0.000	0.507	493.128	624.869	838.101
LF11	Lervik	0.000	0.526	12.986	6.155	10.142	0.000	0.504	493.523	637.649	838.524
LM1	Lervik	0.000	0.520	20.969	8.665	14.624	0.000	0.516	508.703	640.167	863.299
LM10	Lervik	0.000	0.520	19.962	7.946	13.892	0.000	0.505	534.552	614.659	832.266
LM2	Lervik	1.125	0.014	15.996	10.443	9.318	0.000	0.509	539.388	690.057	916.041
LM6	Lervik	0.000	0.516	16.802	8.525	14.667	0.000	0.508	522.995	657.725	889.125
LM7	Lervik	0.000	0.522	19.516	7.821	12.024	0.000	0.501	329.455	420.402	510.549

ID	Population origin	Microsatellites for subset of individuals					SNPs for subset of individuals				
		**-LOG (LH/Lmax)**	**Probability**	**Harfjärden -Log(L)**	**Lervik -Log(L)**	**Oknebäck -Log(L)**	**-LOG (LH/Lmax)**	**Probability**	**Harfjärden -Log(L)**	**Lervik -Log(L)**	**Oknebäck -Log(L)**
OF4	Oknebäck	0.000	0.511	14.594	10.495	9.911	0.000	0.500	641.932	517.955	879.23
OF5	Oknebäck	0.000	0.515	12.842	11.883	7.488	0.000	0.500	618.267	507.677	847.7
OF8	**Oknebäck**	**1.864**	**0.003**	21.596	*12.556*	14.419	0.000	0.500	675.455	533.983	927.566
OF9	Oknebäck	0.000	0.518	20.384	15.426	9.722	0.000	0.500	636.488	512.549	852.802
OF11	Oknebäck	0.000	0.516	16.695	12.051	7.729	0.000	0.500	597.874	496.226	807.54
OM1	Oknebäck	0.000	0.514	13.626	9.061	7.483	0.000	0.500	587.497	496.097	803.816
OM2	Oknebäck	0.000	0.512	18.453	14.088	10.910	0.000	0.500	660.463	505.795	864.712
OM7	Oknebäck	0.009	0.020	22.161	12.673	12.682	0.000	0.500	627.66	503.3	840.227
OM8	Oknebäck	0.000	0.511	20.278	13.029	11.724	0.000	0.500	598.863	480.493	839.364
OM10	Oknebäck	0.000	0.512	22.929	11.399	8.732	0.000	0.500	612.372	503.928	807.688

Table S7. Outlier SNPs/loci under selection from LFMM (salinity and temperature as environmental variables).

Genomic accession	Position	Linkage group	Gene	Environmental correlation
NC_025977.3	25209337	LG10	zinc finger protein 436-like	temperature
NC_025977.3	24790291	LG10	zinc finger protein 436-like	temperature
NC_025974.3	24790443	LG07	-	salinity
NC_025986.3	11458289	LG19	SH3 and multiple ankyrin repeat domains protein 2	salinity and temperature

References

Catchen, J., Hohenlohe, P. A., Bassham, S., Amores, A., & Cresko, W. A. (2013). Stacks: An analysis tool set for population genomics. *Molecular Ecology, 22*(11), 3124-3140. doi:10.1111/mec.12354

Catchen, J. M., Amores, A., Hohenlohe, P., Cresko, W., & Postlethwait, J. H. (2011). Stacks: Building and genotyping loci de novo from short-read sequences. *G3: Genes|Genomes|Genetics, 1*(3), 171-182. doi:10.1534/g3.111.000240

Chapuis, M.-P., & Estoup, A. (2006). Microsatellite null alleles and estimation of population differentiation. *Molecular Biology and Evolution, 24*(3), 621-631. doi:10.1093/molbev/msl191

Paris, J. R., Stevens, J. R., & Catchen, J. M. (2017). Lost in parameter space: A road map for stacks. *Methods in Ecology and Evolution, 8*(10), 1360-1373. doi:10.1111/2041-210X.12775

Rochette, N. C., & Catchen, J. M. (2017). Deriving genotypes from RAD-seq short-read data using Stacks. *Nature Protocols, 12*, 2640. doi:10.1038/nprot.2017.123
